# Characterization of the immune profile of oral tongue squamous cell carcinomas with advancing disease

**DOI:** 10.1002/cam4.3106

**Published:** 2020-05-08

**Authors:** Katie Meehan, Connull Leslie, Michaela Lucas, Angela Jacques, Bob Mirzai, James Lim, Max Bulsara, Yasir Khan, Nicholas C. Wong, Benjamin Solomon, Chady Sader, Peter Friedland, Gisela Mir Arnau, Timothy Semple, Annette M. Lim

**Affiliations:** ^1^ Chinese University of Hong Kong Hong Kong; ^2^ Department of Anatomical Pathology PathWest Laboratory Medicine Queen Elizabeth Medical Centre Nedlands WA Australia; ^3^ University of Western Australia Crawley WA Australia; ^4^ Department of Clinical Immunology PathWest and Sir Charles Gairdner Hospital Queen Elizabeth Medical Centre Nedlands WA Australia; ^5^ Institute for Health Research University of Notre Dame Fremantle WA Australia; ^6^ Department of Research Sir Charles Gairdner Hospital Nedlands WA Australia; ^7^ Genomics WA Telethon Kids Institute West Perth WA Australia; ^8^ Department of Medical Oncology Sir Charles Gairdner Hospital Nedlands WA Australia; ^9^ Monash Bioinformatics Platform Monash University Clayton VIC Australia; ^10^ Peter MacCallum Cancer Centre Melbourne VIC Australia; ^11^ Department of Oncology Sir Peter MacCallum The University of Melbourne Melbourne VIC Australia; ^12^ Department of Otolaryngology, Head and Neck Skull Base Surgery Sir Charles Gairdner Hospital Nedlands WA Australia; ^13^ School of Medicine University of Notre Dame Fremantle WA Australia

**Keywords:** FOXP3, Immune signature, oral tongue squamous cell carcinoma, PD‐L1

## Abstract

We investigated whether a unique immune response was instigated with the development of oral tongue squamous cell carcinomas (OTSCC), with/without nodal involvement, with/without recurrent metastatic disease, or within tumor involved nodes. One hundred and ten formalin‐fixed paraffin‐embedded samples were collected from a retrospective cohort of 67 OTSCC patients and 10 non‐cancerous tongue samples. Targets including CD4, CD8, FOXP3, PD‐L1, and PD‐1 were analyzed by immunohistochemistry. The Nanostring PanCancer Immune Profiling Panel was used for gene expression profiling. Data were externally validated in the The Cancer Genome Atlas (TCGA) head and neck (HNSCC), melanoma and lung squamous cell carcinoma (LSCC) cohorts. A 24‐immune gene signature was identified that discriminated more aggressive OTSCC cases, and although not prognostic in HNSCC was associated with survival in other TCGA cohorts (improved survival for melanoma, *P* < .001 and worse survival for LSCC, *P* = .038). OTSCC exhibited concordant gene and immunohistochemical (IHC) features characterized by a TH‐2 biased, proinflammatory profile with upregulated B cell and neutrophil gene activity and increased CD4, FOXP3, and PD‐L1 expression (*P* < .001 for all by IHC). Compared to less advanced disease, nodal involvement and recurrent OTSCC did not induce a different immune response although recurrent disease was characterized by significantly higher PD‐L1 expression (*P* = .004 by SP263, *P* = .013 by 22C3, *P* = .004 for gene expression). Identification of a gene signature associated with different prognostic effects in other cancers highlights common pathways of immune dysregulation that are impacted by the tumor origin. The significant immunosuppressive signaling in OTSCC indicates primary failure of immune system to control carcinogenesis emphasizing the need for early, combination therapeutic approaches.

## Graphical abstract

Oral tongue squamous cell carcinomas (OTSCC) exhibit primary immune failure to control carcinogenesis demonstrated by the absence of an immune response to the development of nodal metastases or recurrent disease. Proinflammatory immune gene expression is counteracted by immunosuppressive signaling mediated by FOXP3 and PD‐L1 expression. A gene signature was identified that was not prognostic in HNSCC but was significantly associated with survival in other cancers, demonstrating histotype‐dependent prognostic effects of immune regulation.

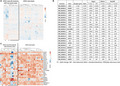

**Abbreviations**
CI: confidence interval
FC: fold change
FDR: false discovery rate
FFPE: formal‐fixed paraffin‐embedded
H&E: hematoxylin and eosin
HNSCC: head and neck squamous cell carcinoma
HPV: human papillomavirus
HR: hazard ratios
IHC: immunohistochemical
Intra: intratumoral
LSCC: lung squamous cell carcinoma
ND: not detected
NK: Natural Killer (cells)
OR: odds ratio
ORR: overall response rate
OTSCC: oral tongue squamous cell carcinoma
Peri: peritumoral
QC: quality control
StDev: standard deviation
TCGA: The Cancer Genome Atlas
TPS: tumor positive score
Treg: T regulatory (cells)

## 1 INTRODUCTION

Head and neck squamous cell carcinoma (HNSCC) refer to a heterogeneous group of mucosal cancers encompassing those arising from the oral cavity, oropharyngeal, hypopharyngeal, and laryngeal subsites. The success of immune checkpoint inhibitors for the treatment of recurrent metastatic HNSCC has highlighted the role of the tumor microenvironment in carcinogenesis.^1^, ^2^ However, the modest overall response rates (ORR) of up to 23% for single agent immunotherapy suggests that evasion of multiple immunomodulatory checkpoints is contributory to the disease development. Indeed, impaired immunity is known to be relevant in the pathogenesis of HNSCC through both a defective T cell response and presence of inhibitory cytokines.^3^, ^4^ Despite knowledge that unique clinical and molecular features exist according to each HNSCC subsite,^5^, ^6^, ^7^ the existence of unique subsite‐specific immunomodulatory mechanisms is unknown due to the study of heterogeneous cohorts. For example, while the human papillomavirus (HPV) accounts for the increasing number of oropharyngeal cancers,^8^ the reason for the increasing incidence of oral tongue squamous cell carcinomas (OTSCC), the most common cancer arising from the oral cavity subsite, is not understood.

Recent studies have contributed information regarding the relevance of the immune system in HNSCC. Analyzes of 280 heterogeneous HNSCC tumors from The Cancer Genome Atlas (TCGA) identified HNSCC as highly enriched for Natural Killer (NK) and T regulatory (Treg) cells, and that varying levels of immune infiltration and activation were present when samples were defined according to molecular subtypes, HPV status, or cigarette exposure.^9^ Notably, a variety of immune characteristics correlated differentially with patient survival. The most well‐known immune gene signature predictive of response to immunotherapy targeting the PD‐1/PD‐L1 checkpoint and prognostic of a favorable outcome is the T cell‐inflamed gene expression profile.^10^, ^11^, ^12^ The six gene signature (*IDO1*, *CXCL10*, *CXCL9*, *HLA‐DRA*, *STAT1*, and *IFNG*) and expanded 18‐gene signature predict response to immunotherapy for HNSCC patients and those with other cancers.^10^, ^12^ Recently, Chen et al interrogated the TCGA HNSCC data and identified the less common, better prognosis “Active Immune Class” and the poor prognosis “Non‐Immune Class” (*P* = .03 for overall survival).^13^ Patients with the T cell‐inflamed signature grouped within the “Active Immune Class” which was associated with oropharyngeal tumors (*P* < .001) and the presence of HPV (*P* < .001). Data according to subsite was not reported but the majority of oral cavity tumors which include OTSCC, were found within the poor prognosis “Non‐Immune Class.” Of note, some early data suggest that immunotherapy response may vary according to subsite with oral cavity tumors associated with an increased risk of early progression,^14^ and that HNSCC response to immunotherapy may be discordant between primary and nodal disease defined by an absence of a “TH‐1 response”.^15^ It is likely that while common immune pathways are apparent in heterogeneous HNSCC cancers, unique immune characteristics according to tumor subsite and tumor location (primary vs nodal) are also likely to exist.

Therefore, we sought to determine if a unique immune response was instigated in OTSCC with advancing disease and if this impacted on patient outcome. We report here the immune characteristics of (a) OTSCC compared to non‐cancerous oral tongue samples; (b) node positive OTSCC vs OTSCC without nodal involvement; (c) recurrent metastatic OTSCC vs non‐recurrent disease; and (d) primary OTSCC vs tumor in matched, involved lymph nodes. Formalin‐fixed paraffin‐embedded (FFPE) samples were assessed using expression array analysis by Nanostring and immunohistochemical (IHC) techniques.

## 2 MATERIALS AND METHODS

### 2.1 Patient data

This study included 110 samples from 67 OTSCC cases and 10 non‐cancer cases. The OTSCC cases included 32 cases without nodal involvement, 18 cases with nodal involvement and 17 cases with recurrent metastatic disease. Clinicopathological details are summarized in Table S1. Non‐cancer tongue samples were obtained from a cohort of five males and five females with a median age of 58 (range 53‐67) years who had biopsies of clinically apparent areas but for which no invasive cancer, carcinoma in‐situ or dysplasia was found. Institutional ethics approval was obtained for the conduct of the study.

### 2.2 RNA extraction and NanoString analysis

A FFPE section stained with hematoxylin and eosin (H&E) was examined by a head and neck pathologist to confirm the presence of invasive tumor. For RNA purification (High Pure RNA Isolation Kit, Roche Diagnostic), either 2‐4 × 10 μm scrolls were obtained or if the tumor percentage was <70%, macro‐dissection was performed on multiple air‐dried sections. RNA quantity and integrity were determined with the NanoDrop ND‐2000 UV‐Vis Spectrophotometer (Thermo Fisher Scientific) and with either a LabChip GX (PerkinElmer) or a TapeStation (Agilent Technologies). A minimum of 200 ng of RNA from 44 OTSCC and six non‐cancer samples was used to measure the expression of 730 immune‐related genes and 40 housekeeping genes using the nCounter® platform (NanoString Technologies) and the PanCancer Immune Profiling Panel according to the manufacturer's instructions. PD‐1 expression is not assessed by this platform. Briefly, input RNA was hybridized to target sequence‐specific capture probes and fluorescent‐labeled reporter probes for 15‐19 hours at 65°C. The mRNA‐probe complexes were washed, immobilized, and quantified by the nCounter digital analyzer according to manufacturer's instructions.

### 2.3 Immunohistochemistry

Immunohistochemical staining was performed on whole sections using the BenchMark ULTRA platform (Roche) for all stains except the PD‐L1 clone 22C3 which was performed on the Autostainer Link 48 (Dako). If NanoString RNA analysis was performed on a case, the identical FFPE tissue block was used for IHC staining. 4 µm sections were cut, mounted on adhesive slides (Trajan® Series 3), dewaxed and rehydrated using xylene and graded alcohol washes then dried in an oven at 60°C for 60 minutes. Antigen retrieval and blockage of endogenous peroxidase activity was performed as per manufacturer's instructions. Primary antibody dilution was as per manufacturer's instructions and as validated by PathWest Laboratory Medicine as follows; CD3 (polyclonal, 1:600; Dako), CD4 (SP35, pre‐dilute; Cell Marque), CD8 (144B, 1:50; Dako), CD56 (1B6, 1:50; Leica), FOXP3 (SP97, 1:200; Spring Bioscience), PD‐1 (NAT105, pre‐dilute; Ventana), and PD‐L1 (SP263, pre‐dilute, Ventana) with the reaction was visualized using the Ventana 3 step detection system OptiView (cat no. 950‐224). Staining for the PD‐L1 clone 22C3 was performed using the prediluted pharmDx kit (SK006, Dako) as per manufacturer's instructions. Background staining was performed with Mayers’ hematoxylin. Negative and positive controls were included (benign tonsil, and in addition placenta for PD‐L1 clones).

Slides were scanned with a ScanScope XT (Aperio) digital microscope slide scanner (Olympus UPlanSApo 20x NA0.75 objective). Visualization and image analysis assessment was carried out using Tissue Image Analysis, version 3.0 (Slidepath). Visual assessment of stained slides was performed prior to digital image analysis.

Assessment of both PD‐L1 clones was undertaken manually (Olympus BX51). The representative tumor section was scored as a tumor proportion score (TPS) by a Pathologist experienced in PD‐L1 scoring, using the method recommended for companion diagnostic testing in non‐small cell lung carcinoma.^16^ In brief, positive staining was considered any perceptible linear cell membrane staining (partial or complete) in viable tumor cells, excluding any associated immune cells, benign cells, cytoplasmic staining, and necrotic areas. The percentage of positive staining tumor cells of the total assessable tumor cells was scored. As results were similar between the PD‐L1 clones (Table S2) these are only reported as one entity, unless specified.

Prior to image analysis, representative intra‐ and peritumoral regions were annotated by a Pathologist (Figure S1) and subsequently submitted for batch image analysis. Cytoplasmic and nuclear image analysis algorithms were optimized and deployed within SlidePath's Tissue Image Analysis system, a web‐enabled image analysis solution for the interpretation of virtual slides. A nuclear algorithm was used for FOXP3 and a cytoplasmic algorithm was used for CD3, CD4, CD8, CD56, and PD‐1 (at the resolution scanned immunohistochemical staining in lymphocytes cannot differentiate membranous or cytoplasmic staining). The output from the algorithm was reported as the total number of positive cells per mm^2^ including quantitative measurements of cytoplasmic or nuclear staining absorbance, total tissue area (mm^2^), and the total number of positive cells counted in the area. As results for intra‐ and peritumoral regions were similar these are reported as one entity, unless specified. For non‐cancer cases, the “peripheral” or “peri‐target” areas refer to stroma immediately adjacent to thickened (but not neoplastic) epithelium.

### 2.4 NanoString data analysis

Reporter probe counts for samples were subjected to sequential data‐processing steps, starting with quality control (QC) metrics using the NSolver software v4.0 (NanoString Technologies). Four samples that failed QC metrics were excluded. Remaining samples were normalized and differential expression analyzes were performed using three different software packages (Table S3; Data Files [Link], [Link], [Link], [Link]; NanoStringDiff, R v3.3.3, http://www.r‐project.org ; Degust v3.1, https://degust.erc.monash.edu/ ; NSolver Advanced Analysis module v2.0.115).^17^, ^18^ Significant differential gene expression was determined by a false discovery rate (FDR) of *q* < 0.05 and log2 fold change (FC) >1.5 by at least two software packages. Hierarchical clustering analysis was based on Euclidean distance and Ward's minimum variance method with two‐group (*k* = 2) clustering. Heatmaps were generated using NanoStringNorm (R v3.3.3) normalized data with ClustVis.^19^ Gene networks were visualized using STRING v10.0 and biological processes were analyzed using the Panther classification system.^20^, ^21^

### 2.5 Statistical analyzes

Patient demographics and tumor attributes were described and compared by independent sample Student's *t* test or Pearson's or Fisher Exact *χ* ^2^ test, as appropriate. Time to death was estimated using Kaplan‐Meier survival probabilities, with log‐rank tests used to test statistical differences between survival curves for cancer signature strata (downregulated and/or upregulated genes signature). Cox proportional hazards regression modeling was used to generate corresponding hazard ratios (HRs) and 95% confidence intervals (95% CI). Logistic regression was used to identify gene combinations that associated with mortality. Statistical analysis was conducted using Stata 15.0 (StataCorp LLC) and SPSS version 24.0 (Armonk). All hypothesis tests were two‐sided, and *P* < .05 were considered statistically significant. Additional analyzes are summarized in Table S4.

## 3 RESULTS

### 3.1 OTSCC cases relative to non‐cancer cases

#### 3.1.1 Immune gene expression profiling

The immune gene expression profile of 40 OTSCC samples (tumor only samples from 14 node negative cases, 14 node positive cases and 12 cases with recurrent disease) and six non‐cancer cases were analyzed.

**FIGURE 1:**
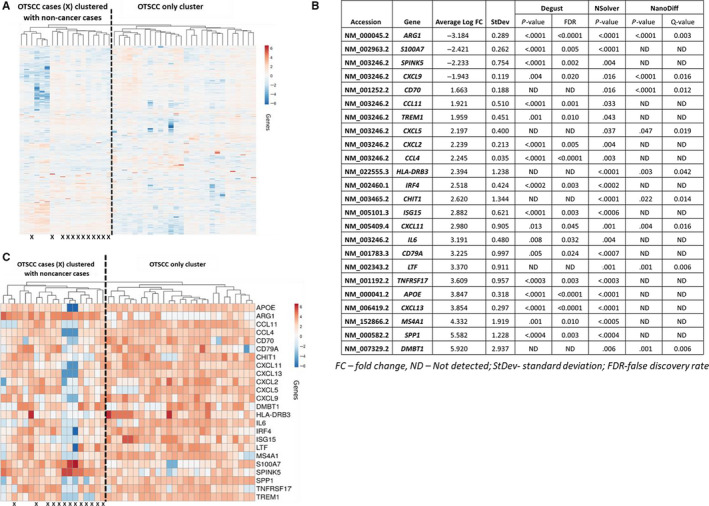
Summary of oral tongue squamous cell carcinomas (OTSCC) vs non‐cancer analysis. A, Unsupervised hierarchical cluster analysis of OTSCC vs non‐cancer cases according to the log2 fold change of gene expression. B, Differentially expressed genes in OTSCC relative to non‐cancer cases. Genes that were identified as differentially expressed >1.5 log fold change by 2 or more data analyses packages were included. C, Supervised hierarchical cluster analysis of OTSCC vs non‐cancer cases according to the log2 fold change of gene expression

Unsupervised hierarchical clustering revealed that 12 OTSCC samples grouped with the non‐cancer cases (Figure 1A). The 28 OTSCC cases that did not cluster with the non‐cancer cases represented a subgroup with significantly more aggressive histopathological features and were significantly larger in pathological size (*P* = .013), more likely to have nodal involvement (*P* < .001) and greater depth of invasion (*P* = .006).

Twenty‐four genes were significantly differentially expressed in OTSCC compared to non‐cancer cases consistent with a proinflammatory phenotype (Figure 1B). Supervised hierarchical clustering analysis using these 24 differentially expressed genes revealed that 27 OTSCC cases clustered together vs those that clustered with non‐cancer cases (Figure 1C). Correlative statistical analyzes revealed that the 27 OTSCC cluster were larger in size (*P* = .012), more likely to have nodal involvement (*P* = .005), and greater depth of invasion (*P* = .008). Although not significant (*P* = .155), the 13 patients whose samples clustered with non‐cancer cases were alive and disease free at the time of data censoring except for three patients who died of other noncancer‐related causes. Furthermore, subsequent pathological review of the OTSCC H&E slides blinded to the clustering analyzes revealed that the 24‐immune gene signature selected the majority of tumors (13/18) with ulceration evident. Thus, the 24‐immune gene signature defined a subgroup of 27 samples with more aggressive histopathological features.

The ability of the 24‐gene signature to predict clinical outcome was firstly validated in silico using the TCGA HNSCC (n = 542), melanoma (n = 472), and lung squamous cell carcinomas (LSCC, n = 551) cohorts that had both RNAseq data and survival data available (https://portal.gdc.cancer.gov/, download date 4 April 2019). A priori the LSCC TCGA cohort was selected due to the similarities in risk factors and histotype with OTSCC, and the melanoma cohort was chosen due to the differences between diseases. Patient survival was compared according to the presence of the full cancer signature, part of the cancer signature or no cancer signature, and combinations of these groupings. Analyzes were limited to only 23 genes as *HLA‐DRB3* was not examined in TCGA transcriptome analyzes.

For the TCGA HNSCC data analyzes, “Lip, NOS” cases were excluded to ensure only mucosal carcinomas were examined. Initial analyzes were performed on a randomly selected test cohort (n = 277), followed by the full HNSCC cohort (n = 542) and the OTSCC subgroup (annotated as “border of tongue” and “Tongue, NOS,” n = 139). In the test cohort, the presence of the 23 immune gene signature was associated with shorter median overall survival (OS) being 18 months for those with the full cancer signature (HR = 2.67, 95% CI 1.55‐4.61, *P* < .001) vs 40 months for those with part of the signature (HR = 1.45, 95% CI 0.88‐2.37, *P* = .137) relative to 90 months for those without the signature (Table 1A). However, in the full HNSCC cohort the presence of the signature was not significantly associated with OS (52 months for both, for the presence of the full or part cancer signature vs no signature, respectively; *P* = .289; Table 1B). Similarly, in the OTSCC subgroup the presence of the signature was not associated with OS (*P* = .356, Table 1C).

**TABLE 1:** Survival and hazard analyses stratified by the presence of the cancer signature for (A) Test TCGA HNSCC cohort (n = 277); (B) Full HNSCC TCGA cohort (n = 542); and (C) OTSCC TCGA subgroup (n = 139). Logistic regression models* of predictor genes on outcome (death) using the full HNSCC TCGA cohort (D) and the OTSCC subgroup (E). *Additional statistical details are in Table S5

Signature	N	N deaths	Median months	*P*‐value (Log rank)	HR	95% CI HR	*P*‐value (Cox)
(A) Test TCGA HNSCC cohort							
No	71	24	90	.001	1.00		
Part	142	58	40		1.45	0.88‐2.37	.137
Full	64	34	18		2.67	1.55‐4.61	.001
No	71	24	90	.019	1.00		
Part or Full	206	92	30		1.74	1.09‐2.77	.21
(B) Full HNSCC TCGA cohort							
No	130	69	52	.568	1.00		
Part	277	125	55		0.86	0.64‐1.15	.309
Full	135	57	48		0.87	0.61‐1.23	.422
No	130	69	52	.289	1.00		
Part or Full	412	182	52		0.86	0.65‐1.14	.289
(C) OTSCC TCGA subgroup							
No	54	26	37	.435	1.00		
Part	62	26	90		0.75	0.39‐1.45	.391
Full	23	9	52		0.74	0.31‐1.77	.502
No	54	26	37	.197	1.00		
Part or Full	85	35	90		0.75	0.41‐1.38	.356

Gene	Univariate models			Multivariable model #1			Final multivariable model		
	OR	95% CI OR	*P*‐value	OR	95% CI OR	*P*‐value	OR	95% CI OR	*P*‐value
(D) Logistic regression modeling for Full HNSCC TCGA cohort									
*ARG1*	1.00	0.94‐1.06	.949						
*S100A7*	0.99	0.94‐1.04	.667						
*SPINK5*	0.98	0.91‐1.05	.563						
*CXCL9*	0.95	0.89‐1.02	.153						
*CD70*	0.99	0.92‐1.06	.718						
CCL11*	0.92	0.86‐0.97	.005	0.92	0.82‐0.96	.002	0.92	0.82‐0.98	.016
*TREM1*	0.98	0.90‐1.05	.525						
*CXCL5*	0.97	0.92‐1.02	.292						
*CXCL2*	1.00	0.91‐1.09	.934						
*CCL4*	0.94	0.84‐1.04	.206						
IRF4*	0.86	0.78‐0.94	.001						
*CHIT1*	0.97	0.92‐1.02	.205						
ISG15*	0.92	0.85‐1.00	.050						
*CXCL11*	0.97	0.92‐1.03	.303						
***IL6***	1.06	0.99‐1.14	.104	1.14	1.05‐1.24	.002	1.14	1.05‐1.24	.001
***CD79A*** *	0.87	0.82‐0.93	<.001	0.88	0.82‐0.94	<.001	0.88	0.82‐0.95	.001
*LTF*	0.98	0.94‐1.02	.395						
TNFRSF17*	0.89	0.83‐0.94	<.001						
APOE**	1.00	0.92‐1.09	.951	1.13	1.01‐1.25	.026			
CXCL13*	0.90	0.84‐0.97	.004						
MS4A1*	0.90	0.85‐0.95	<.001						
*SPP1*	1.04	0.98‐1.11	.220						
*DMBT1*	0.99	0.96‐1.03	.753						

Gene	Univariate models			Final multivariable model		
	OR	95% CI OR	*P*‐value	OR	95% CI OR	*P*‐value
(E) Logistic regression modeling for TCGA OTSCC subgroup						
*ARG1*	1.01	0.90‐1.14	.837			
*S100A7*	0.96	0.86‐1.07	.467			
*SPINK5*	0.99	0.85‐1.15	.842			
*CXCL9*	0.98	0.86‐1.11	.727			
*CD70*	1.01	0.87‐1.18	.870			
***CCL11*** *	0.88	0.78‐1.00	.051	0.81	0.70‐0.94	.005
*TREM1*	0.92	0.80‐1.07	.293			
*CXCL5*	0.98	0.89‐1.08	.690			
*CXCL2*	1.06	0.90‐1.25	.490			
*CCL4*	0.98	0.79‐1.22	.866			
*IRF4*	0.91	0.75‐1.11	.367			
*CHIT1*	0.96	0.86‐1.08	.516			
*ISG15*	0.96	0.82‐1.12	.576			
*CXCL11*	1.02	0.91‐1.14	.749			
IL6**	1.08	0.94‐1.23	.276	1.21	1.03‐1.42	.021
*CD79A*	0.92	0.80‐1.07	.288			
*LTF*	1.06	0.96‐1.16	.265			
*TNFRSF17*	0.94	0.83‐1.06	.315			
*AP* *OE*	0.97	0.81‐1.16	.729			
CXCL13*	0.87	0.75‐1.00	.054			
MS4A1*	0.90	0.81‐1.00	.059			
*SPP1*	0.98	0.87‐1.11	.769			
*DMBT1*	1.04	0.95‐1.14	.379			

Abbreviations: CI, confidence interval; HR, hazard ratio; N, number; OR, odds ratio.

Of the 24 genes in the cancer signature, multiple logistic regression modeling revealed that only three genes (*CCL11*, *CD79A*, and *IL6*) were significantly associated with the outcome of death in the TCGA HNSCC cohort (Table 1D). High *IL6* expression was associated with higher odds of death in both the HNSCC cohort (HR = 1.14, 95% CI 1.05‐1.24, *P* = .001) and OTSCC subgroup (HR = 1.21, 95% CI 1.03‐1.42, *P* = .021, Table 1E). Similarly, high *CCL11* expression was associated with lower odds of death for the HNSCC cohort (HR = 0.92, 95% CI 0.82‐0.98, *P* = .016) and OTSCC subgroup (HR = 0.81, 95% CI 0.70‐0.94, *P* = .005). High *CD79A* expression was associated with lower odds of death for HNSCC only (HR = 0.88, 95% CI 0.82‐0.95, *P* = .001). However, combinations of these three genes were not able to dichotomize survival in the HNSCC or OTSCC cohorts.

**FIGURE 2:**
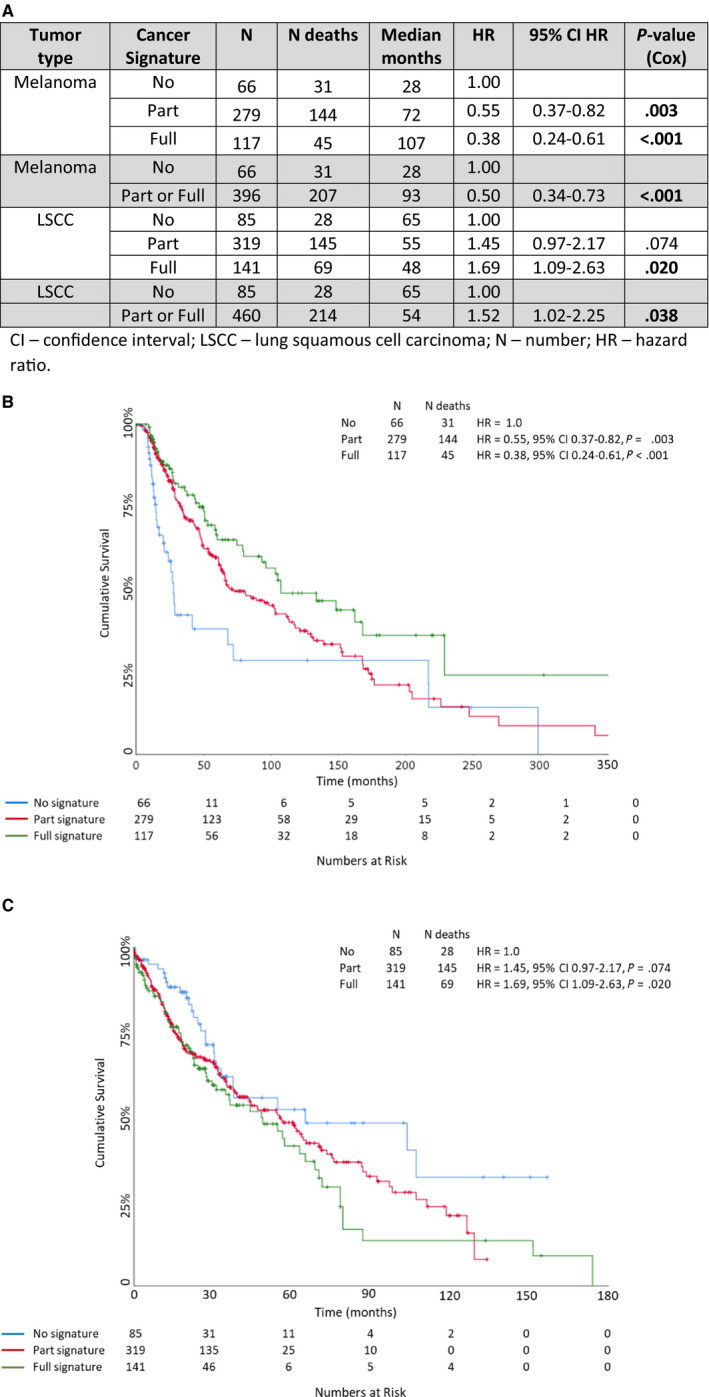
A, Patient survival according to the presence of the cancer signature in the The Cancer Genome Atlas (TCGA) melanoma and lung squamous cell carcinoma (LSCC) cohorts. Kaplan‐Meier curves for the B, TCGA melanoma cohort (n = 472), and C, TCGA LSCC cohort (n = 551) according to the presence of the full cancer signature vs part of the cancer signature and no cancer signature

Of interest, in the TCGA melanoma cohort the 23 immune gene signature was associated with significantly improved OS. The signature proportionally stratified OS with median OS of 28 vs 72 vs 107 months for those without, with part of the signature, and with the full signature, respectively (HR = 0.55, 95% CI 0.37‐0.82, *P* = .003 for part of the signature and HR 0.38, 95% CI 0.24‐0.61, *P* < .001 for the full signature; Figure 2A,B). For the TCGA LSCC cohort the signature conversely was associated with significantly worse OS. Again, the signature proportionally stratified OS according to the presence of the full signature vs part of the cancer signature vs no signature with median OS of vs 48 vs 55 vs 65 months, respectively (HR = 1.69, 95% CI 1.09‐2.63, *P* = .020 for the full signature and HR1.45, 95% CI 0.97‐2.17, *P* = .074 for part of the signature, Figure 2A,C). Logistic regression revealed that the key genes responsible for these results were *CXCL9* and *SPP1* for melanoma and *IL6* and *CXCL13* for LSCC. Therefore, the most significantly dysregulated immune genes in a homogeneous OTSCC cohort were found to differentially correlate with survival in two large cohorts of other cancers.

Given these findings, Kaplan‐Meier Plotter (access date 29/4/2019, kmplot.com) was used for further analyzes which curates publicly available gene expression datasets from cancer patients with survival data from the TCGA, publications, and other sources.^22^ Using a FDR of *q* < 0.05, analyzes revealed that high *IL6* expression was associated with higher odds of death for renal clear cell carcinoma (n = 530, HR = 2.49, 95% CI 1.78‐3.49, *P* < .001), as was high *CCL11* (HR = 1.91, 95% CI 1.41‐2.60, *P* < .001). Conversely, high expression of *CD79A* was associated with higher odds of survival in thymomas (n = 118, HR = 0.11, 95% CI 0.03‐0.46, *P* < .001). When the genes were combined, a significantly higher odds of death in renal clear cell carcinoma was confirmed (Figure S2). Therefore, the genes correlating with survival identified by logistic regression in a homogenous cohort of OTSCC were again found to be relevant in other cancer types, and again were associated with a differential impact on survival.

#### 3.1.2 Immunohistochemical profiling and additional combined analyzes

Immunohistochemistry was performed in 67 OTSCC patient samples (primary only tumor samples from 32 node negative cases, 18 node positive cases and 17 cases with recurrent disease) and 10 non‐cancerous oral tongue samples with sufficient tissue for analyzes.

**FIGURE 3:**
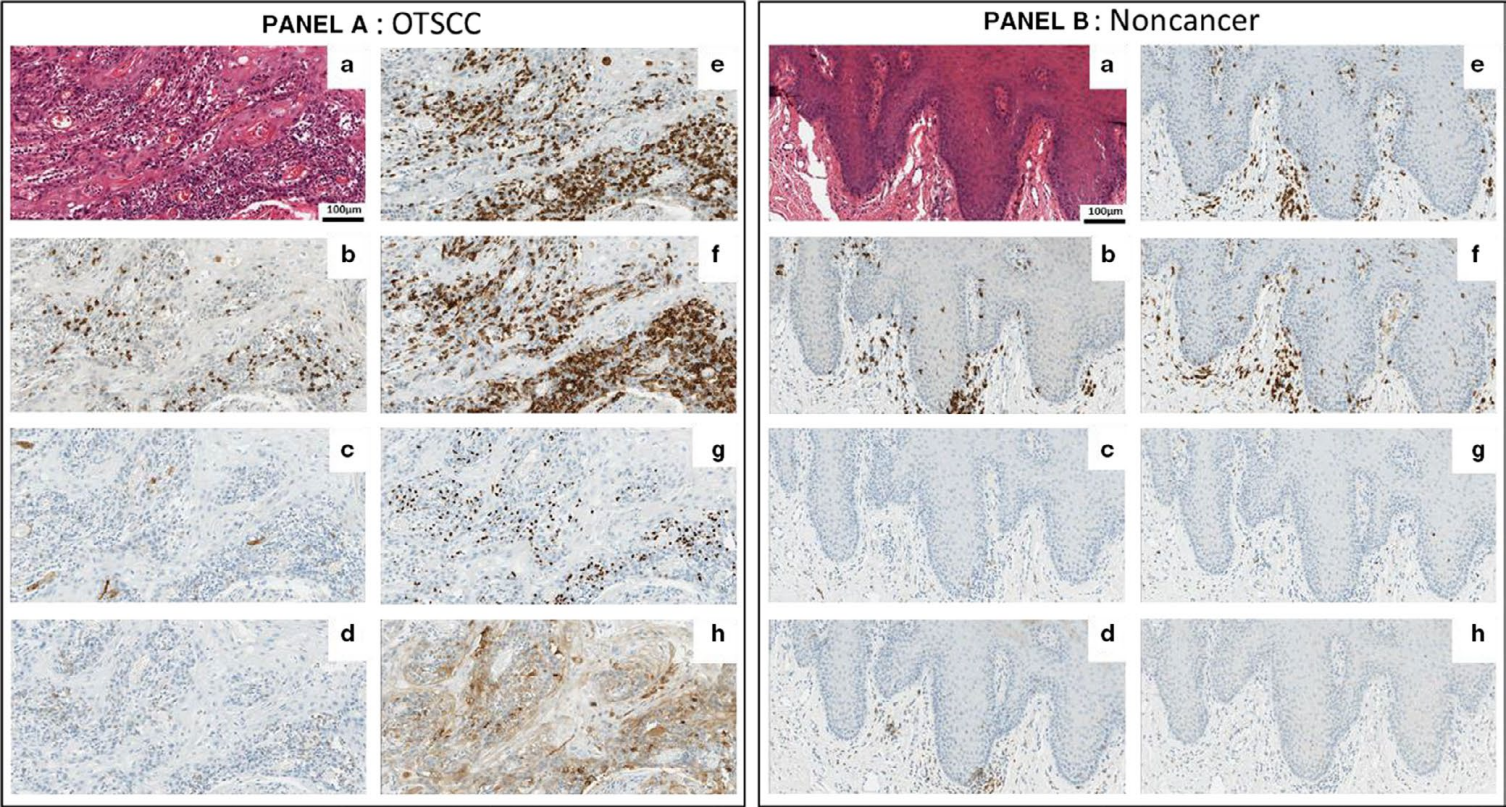
Representative images of immune expression markers in oral tongue squamous cell carcinomas (OTSCC) and non‐cancer cases. Histology for OTSCC (Panel A) and non‐cancer cases (Panel B) assessed by H&E A, with similar expression between OTSCC and non‐cancer cases for CD8 B, CD56 C, and PD‐1 D, and increased expression in OTSCC for CD3 E, CD4 F, FOXP3 G and PD‐L1 assessed by SP263 Ventana antibody H

Expression of CD3, CD4, FOXP3, and PD‐L1 TPS was significantly higher in OTSCC cases relative to non‐cancer cases (Figure 3; Table 2A). However, CD8, CD56, and PD‐1 expression was similar. These observations were generally reflected by the gene expression profiling data, with CD3 and FOXP3 expression found to be significantly higher in cancer cases relative to non‐cancer cases (*P* = .006 and *P* = .027, respectively). CD4 gene expression was higher in cancer samples but statistical significance was not reached. PD‐L1 gene expression was not statistically different between groups.

**TABLE 2:** Comparative immunohistochemical profiling reported according to antibody and cohort examined. (A) Oral tongue squamous cell carcinomas (OTSCC) vs non‐cancer cases. (B) OTSCC with nodal involvement vs OTSCC without nodal involvement. (C) Recurrent OTSCC cases vs nonrecurrent OTSCC cases. (D) Primary OTSCC cases vs tumor in matched, involved nodes. Values reported are the number of cells with positive cytoplasmic staining/mm2 except for FOXP3 reported as positive nuclei/mm2. PD‐L1 tumor positive score (TPS) is reported as a percentage

Antibody	Location of staining	OTSCC median (range)	Noncancer median (range)	*P*‐value (Log Rank)
**(A)**				
CD3	Intra	1391 (202‐9276)	102 (38‐1307)	**<.001**
	Peri	4877 (0‐34327)	2979 (417‐6870)	**.047**
CD4	Intra	2097 (24‐16356)	607 (53‐1923)	**<.001**
	Peri	5601 (50‐29491)	4511 (287‐6877)	**.044**
CD56	Intra	194 (0‐9998)	33 (4‐1177)	.400
	Peri	426 (0‐8806)	544 (0‐3581)	.791
CD8	Intra	1139 (30‐11555)	260 (20‐3925)	.210
	Peri	4661 (113‐30459)	2854 (133‐9681)	.051
PD‐1	Intra	106 (3‐4506)	98 (17‐1970)	.567
	Peri	666 (6‐9724)	405 (48‐6386)	.617
FOXP3	Intra	814 (3‐9700)	160 (25‐304)	**<.001**
	Peri	2284 (22‐13952)	1114 (22‐3010)	.054
PD‐L1	SP263	19 (0‐95)	1 (0‐5)	**<.001**
	22C3	14 (0‐90)	0.4 (0‐1)	**<.001**

Antibody	Location of staining	OTSCC With Nodes median (range)	OTSCC without Nodal involvement median (range)	*P*‐value (log rank)
**(B)**				
CD3	Intra	1498 (407‐9276)	690 (202‐6094)	.406
	Peri	4467 (0‐34327)	4184 (928‐22030)	.990
CD4	Intra	2141 (707‐16356)	1371 (24‐11427)	.681
	Peri	5322 (1860‐29491)	4824 (50‐23814)	.895
CD56	Intra	214 (0‐1990)	121 (0‐9998)	.891
	Peri	602 (0‐3440)	508 (0‐2035)	.925
CD8	Intra	2614 (467‐10050)	675 (64‐11555)	.140
	Peri	11 424 (1000‐30459)	3417 (113‐19754)	**.005**
PD‐1	Intra	104 (5‐1994)	130 (3‐4506)	.364
	Peri	589 (6‐4649)	932 (24‐9724)	.072
FOXP3	Intra	1020 (281‐9700)	682 (133‐3239)	.167
	Peri	2626 (426‐13952)	1929 (88‐11111)	.682
PD‐L1	SP263	4 (0‐80)	2 (0‐30)	.071
	22C3	3 (0‐90)	1 (0‐30)	.062

Antibody	Location of staining	Recurrent OTSCC median (range)	Nonrecurrent OTSCC median (range)	*P*‐value (log rank)
**(C)**				
CD3	Intra	1706 (292‐8346)	1189 (202‐9276)	.539
	Peri	6356 (0‐16880)	4184 (0‐34327)	.77
CD4	intra	2958 (433‐8286)	1829 (24‐16356)	.782
	Peri	7166 (3027‐15390)	4997 (50‐29491)	.54
CD56	Intra	211 (00‐2448)	196 (0‐9998)	.555
	Peri	335 (0‐8806)	555 (0‐3440)	.554
CD8	Intra	1088 (30‐5564)	849 (64‐11555)	.41
	Peri	4831 (955‐14563)	4245 (113‐30459)	.307
PD‐1	Intra	52 (27‐1139)	123 (3‐4506)	.599
	Peri	410 (206‐4875)	754 (6‐9724)	.735
FOXP3	Intra	1491 (3‐3314)	746 (133‐9700)	.751
	Peri	2771 (7‐7633)	2055 (88‐13952)	.923
PD‐L1	SP263	33 (0‐95)	12 (0‐80)	**.004**
	22C3	26 (0‐90)	10 (0‐90)	**.013**

Antibody	Location of staining	Primary OTSCC median (range)	Tumor in matched, involved node median (range)	*P*‐value (log rank)
**(D)**				
CD3	Intra	1425 (407‐9276)	1380 (191‐3018)	.277
	Peri	4467 (2633‐34328)	7859 (4106‐19163)	.531
CD4	Intra	2048 (707‐16356)	2294 (526‐4699)	.589
	Peri	4925 (1860‐29491)	6780 (4882‐21767)	.587
CD56	Intra	590 (65‐1990)	184 (55‐370)	**.012**
	Peri	913 (73‐3440)	167 (33‐407)	**.006**
CD8	Intra	771 (112‐5602)	1254 (94‐2730)	.908
	Peri	4073 (548‐30459)	4092 (490‐8257)	.668
PD‐1	Intra	104 (5‐1994)	127 (2‐4423)	.598
	Peri	589 (6‐4649)	425 (77‐8496)	.624
FOXP3	Intra	1020 (281‐9700)	989 (176‐5150)	.437
	Peri	2626 (426‐13951)	1906 (903‐10845)	.645
PD‐L1	SP263	4 (0‐80)	4 (0‐40)	.626
	22C3	3 (0‐90)	1 (0‐50)	.131

Note

Intra = intratumoral; peri = peritumoral for cancer cases and peripheral for noncancer cases.

Given the features of the 24‐immune gene signature, and concordant IHC and gene expression data demonstrating upregulation of FOXP3 expression in OTSCC, additional analyzes were performed to elaborate the nature and impact of the immunosuppressive signaling. In OTSCC samples, significant upregulation of selected inhibitory T cell checkpoints genes *ICOS* (*P* = .015), *TIGIT* (*P* = .041), *CTLA4* (*P* = .003), *CD160* (*P* = .001), *TNFRSF9* (*P* = .014), and *GITR* (*TNFRSF18*, *P* < .001) was observed but not for *TIM3*, *CD39*, *LAG3*, *CD244*, or *OX40* when compared to non‐cancer samples. PD‐L1 TPS below the mean (20%) assessed by the SP263 antibody was associated with a higher odds of death with median survival being 48 months compared with 85 months (HR = 2.0, 95% CI 1.07‐4.23, *P* = .046). However, these findings were not replicated with the 22C3 antibody and when *FOXP3*, *PD‐L1*, and *CTLA4* were analyzed by gene expression array there were no significant correlations with survival possibly due to the measurement of intermingled tumor and immune cells.

### 3.2 OTSCC with nodal involvement relative to cases without nodal involvement

#### 3.2.1 Immune gene expression profiling

Tumor only samples from 14 OTSCC patients with nodal involvement and 14 without nodal involvement were available for analyzes. Overall, immune gene expression profiles were similar. Unsupervised analyzes revealed no discrete clustering. When comparing gene expression between OTSCC cases with nodal involvement relative to those without nodal involvement, complement component 6 (*C6*) was the only gene found to be differentially underexpressed (log fold change of −1.592, *q* < 0.05).

#### 3.2.2 Immunohistochemical profiling and additional analyzes

This comparison involved only primary tumor specimens from 18 OTSCC samples with nodal involvement and 32 OTSCC samples without nodal involvement. Similar to the gene expression analyzes, IHC profiles in OTSCC cases with and without lymph node involvement were similar (Table 2B). However, *FOXP3* gene expression was found to be significantly higher in tumors with nodal involvement vs those without (*P* = .026).

### 3.3 Recurrent OTSCC cases relative to non‐recurrent cases

#### 3.3.1 Immune gene expression profiling

This analysis involved the comparison of 12 specimens from seven patients with recurrent OTSCC and 28 samples from OTSCC patients that did not develop recurrent disease. Overall, immune gene expression profiles were similar. Unsupervised clustering analyzes revealed no discrete clustering. Upon comparison of gene expression profiles between recurrent and non‐recurrent cases, only two genes were significantly differentially expressed (Table S6). *CXCL9* was significantly overexpressed and *DMBT1* was significantly downregulated (*q* < 0.05 for both) in cases with recurrent disease. Subsequent supervised analysis using these two genes failed to resolve recurrent and non‐recurrent cases into discrete groups. No significant relationships between gene expression and clinicopathological data were identified.

#### 3.3.2 Immunohistochemical profiling and additional analyzes

This comparison involved 17 primary tumor samples from OTSCC cases who developed recurrent disease and 50 from OTSCC cases who did not. As with the gene expression analyzes, immune IHC profiles in recurrent OTSCC cases and non‐recurrent cases were generally similar (Table 2C). However, a significantly higher PD‐L1 TPS was found in samples from patients who developed recurrent OTSCC compared to those who did not (33% [range 0%‐95%] vs 12% [range 0%‐80%], *P* = .004 by SP263, Ventana, and 26% [range 0%‐90%] vs 10% [range 0%‐90%], *P* = .013 by 22C3, Dako antibody staining, Figure S3). This significant difference was confirmed in the gene expression profiling data (*P* = .004).

### 3.4 Primary OTSCC samples relative to tumor within matched, involved lymph nodes

#### 3.4.1 Immune gene expression profiling

**FIGURE 4:**
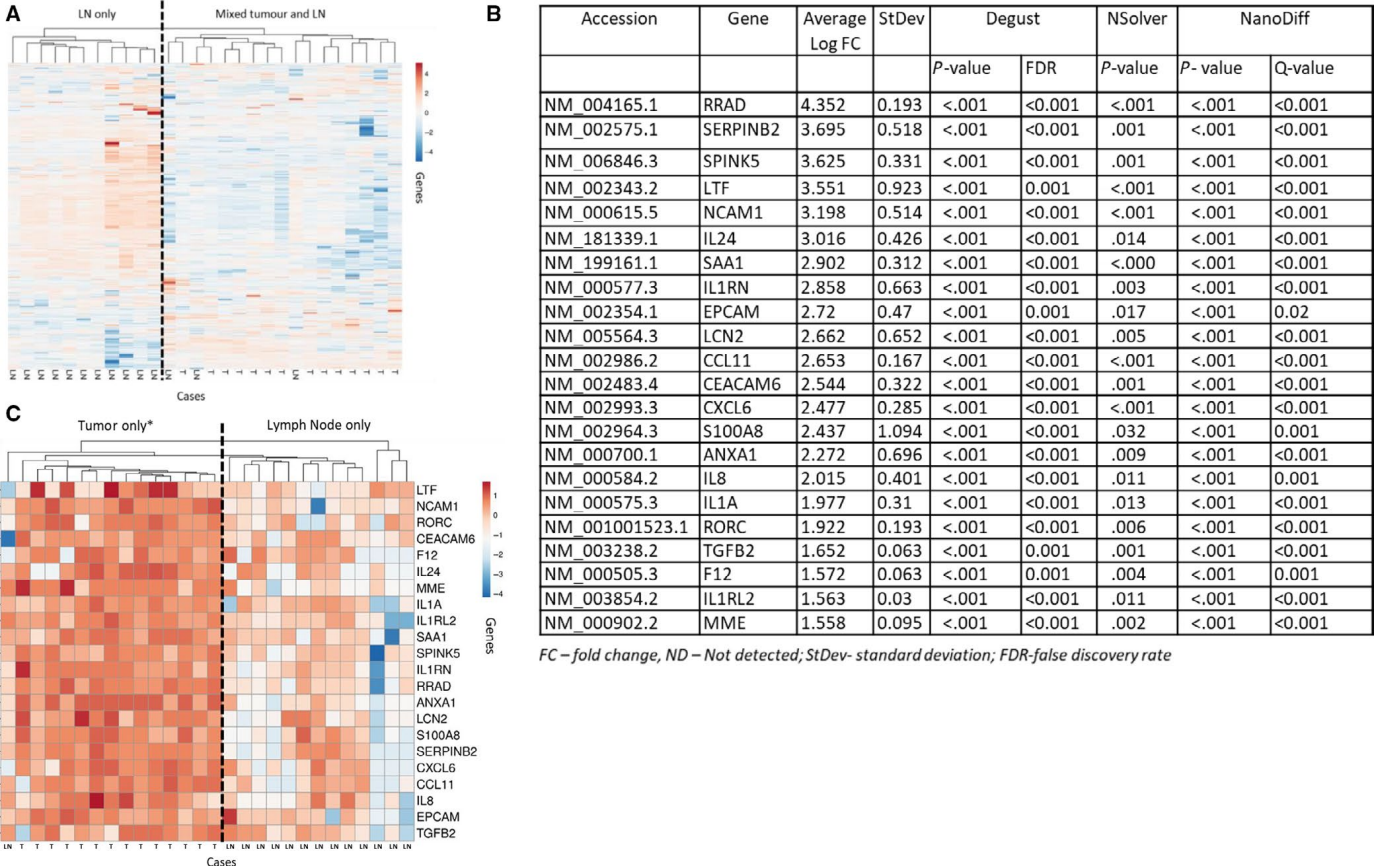
Summary of tumor (T) vs matched lymph nodes (LN). A, Unsupervised hierarchical cluster analysis of tumor and matched, involved lymph nodes from oral tongue squamous cell carcinomas (OTSCC) cases according to the log2 fold change of gene expression. B, Differentially expressed genes in primary tumor compared to matched nodes. C, Supervised hierarchical cluster analysis of tumor (T) and matched, involved LN from OTSCC cases according to the log2 fold change of gene expression (*with the exception of one lymph node sample)

This comparison involved 14 OTSCC samples and tumor from 14 matched, involved lymph nodes. Overall, immune gene expression profiles were different. An unsupervised clustering analysis revealed that primary tumor and nodal disease formed separate clusters with the exception of three cases (Figure 4A). Upon comparison of gene expression between primary tumor samples and tumor within matched nodes, 22 genes were significantly overexpressed in the primary tumor relative to matched lymph node generally representative of the innate immune response (Figure 4B). Subsequent supervised analysis using this 22 gene signature resolved the tumor and matched nodes into two distinct groups with the exception of one nodal sample (Figure 4C).

#### 3.4.2 Immunohistochemical profiling and additional analyzes

This comparison involved 18 primary OTSCC samples with tumor from 18 matched, involved lymph nodes available for analysis. Only expression of CD56 assessed by IHC was significantly higher in the primary tumor compared with the matched lymph node (*P* = .012, Table 2D; Figure S4). However, when assessed by gene expression analyzes, CD3, CD4, and CD56 was significantly upregulated compared to primary tumor (*P* < .001, <.001, and .002, respectively). Other markers (CD8, FOXP3, PD‐1, and PD‐L1) displayed similar expression in the primary tumor and matched lymph nodes.

## 4 DISCUSSION

We investigated whether advancing disease burden in OTSCC precipitated the development of a unique immune response. We identified that OTSCC cases were characterized by a 24‐gene expression profile (GEP) suggestive of an inflammatory phenotype with a TH‐2 type bias. Although not prognostic in HNSCC, the GEP was significantly associated with survival in other cancer cohorts from the TCGA. The signature was associated with longer OS in melanoma and shorter OS in LSCC, highlighting the presence of common immune pathways that exist across cancers with the unsurprising observation that the impact of the immune response differs according to the tumor tissue of origin. Our IHC data confirmed the immunosuppressive features of the GEP, with inhibitory signaling mediated by upregulation of FOXP3 in OTSCC and tumors with nodal involvement. In general, the immunoprofile of OTSCC with clinical features indicative of more aggressive disease (nodal involvement and recurrent disease) did not demonstrate significant immune‐related IHC or gene expression differences from less clinically aggressive disease, beyond upregulated PD‐L1 expression in recurrent disease. Altogether the absence of evolution of the immune response between early and advanced disease suggests that there has been a primary failure of the immune system to control carcinogenesis, and therefore this suggests that OTSCC patients are likely to require combination immunotherapy or chemo‐immunotherapy approaches.

Although effective antitumor immunity plays a crucial role in cancer control, it is well recognized that a chronic inflammatory, immunosuppressed environment can conversely contribute to tumor growth.^23^, ^24^ We identified a 24‐GEP (Figure 1) in OTSCC, collectively representative of the transition away from a CD8 cytotoxic T cell response to a more TH‐2 type, B cell/humoral response with additional upregulation of genes involved in innate immunity and mucosal biology. The GEP consisted of functionally related proinflammatory chemokines (eg, *IL6*, *CD79A*, *CXCL11*; Figure S5) featuring genes that modulate B cell maturation, survival, activation, and humoral immunity (*SPP1*,* MS4A1*, *CXCL13*,* TNFRSF17*, *CD79A*, *IRF4*, *HLA‐DRB3*, *CCL4*). Additionally, given the mucosal origin of OTSCC, the GEP included genes mediating innate (*DMBT1*, *LTF*, *SPINK5*) or mucosal biology (*APOE*, *ISG15*, *IRF4*, *CCL4*, *S100A7*) with modulation of neutrophil (*SPP1*, *ISG15*, *CHIT1*, *CXCL2*, *CXCL5*, *TREM1*) and eosinophil function (*CCL11*). Indeed, 13/18 OTSCC samples with ulceration clustered according to the presence of the GEP, accounting for genes upregulating neutrophil activity. Furthermore, although T cell activation was evident in the GEP, IHC and subsequent gene analyzes suggested that cytotoxic function was likely suppressed through the upregulation of FOXP3, PD‐L1, and CD4 expression.

An interesting observation of our analyzes was that the GEP was not prognostic in the TCGA HNSCC cohort but was prognostically relevant in different cancer types, with the signature correlating with improved survival in melanoma but worse survival in LSCC. Logistic regression analyzes identified that only *IL6* was significantly associated with death within the HNSCC TCGA cohort and OTSCC subgroup, with similar correlation found for increased *IL6* expression in the TCGA LSCC and another external cohort of renal clear cell carcinoma. Conversely, elevated *CCL11* was associated with improved odds of survival in HNSCC and OTSCC subgroup but increased odds of death for renal clear cell carcinoma. The varied prognostic correlation of the immune response in different cancers is not surprising, given that the nature of the immune response ought to vary according to the origin of these tumors (eg, mucosal immunity vs innate immunity). This observation is not dissimilar to the plethora of published discordant predictive and prognostic biomarker data that is desired to be uniform across cancer types, but unsurprisingly is not.^11^ Furthermore, key genes such as *IL‐6* were identified on logistic regression to drive the correlation with survival. An IL‐6 rich microenvironment characterizes the transition between the innate and acquired immunity, toward a T‐helper 2 response with IL‐4 and IL‐10 suppressing cytotoxic CD8 T cell function.^25^, ^26^, ^27^, ^28^ However, while IL‐6 is pro‐tumorigenic supporting cellular proliferation, metastases and survival through mechanisms including activation of the JAK/STAT3 signaling pathways, high levels have been reported to be a poor prognostic marker in cancer.^28^, ^29^ That is, IL‐6 is a pleiotropic cytokine known to also contribute to the development of protective antitumor immunity,^27^ which may account for its differential effect on patient survival in different cancers. Indeed, recent data from large cohorts of immunotherapy‐treated melanoma patients confirm elevated IL‐6 at baseline as a poor prognostic factor.^30^ Altogether, our data support the notion that the immune response to cancer is a dynamic phenomenon that may be both pro‐ and antitumorigenic over time, which may differ according to the anatomical location of the immune response rather than being a variable with a simple binary effect. Therefore, the importance of the identified GEP is the fact that it highlights these common immune pathways which are capable of impacting patient prognosis and may represent potential common targets of therapeutic interest in different cancers.

An interesting component of the 24‐gene signature was that the most downregulated gene was Arginase 1 (*ARG1*). Increased amino acid catabolism, specifically of arginine by ARG1 and tryptophan by Indoleamine 2,3 dioxygenase (IDO), are a hallmark of initial tumorigenesis with the activity of both enzymes known to facilitate immune tolerance.^31^, ^32^ Metabolically, ARG1 depletes extracellular L‐arginine by catalyzing L‐arginine to produce urea and L‐ornithine, starving T lymphocytes of substrate and thus drives an immunosuppressive phenotype. We identified significantly reduced expression of *ARG1* in OTSCC. Downregulation of *ARG1* activity may occur in relationship to p53‐mediated activity suppressing ureagenesis in attempt to hinder tumor growth,^33^ although *TP53* alterations are the most common mutation identified in HNSCC.^7^ Therefore, our observation is likely explained by the known dependence of some cancers such as melanoma, on arginine as a crucial substrate for proliferation and growth (“arginine auxotrophy”) rather than the downregulation of the gene being related to its immunomodulating effect.^34^, ^35^ This suggests that arginine depletion may be relevant as a therapeutic strategy in OTSCC.

Significant upregulation of FOXP3 cells was observed in OTSCC using IHC and gene analyzes, and significantly more FOXP3 expression was found in tumors with nodal involvement compared to those without by gene expression analysis (*P* = .026). Our findings confirm previous reports that demonstrate HNSCC as the most highly infiltrated cancer with regulatory T (Treg) cells.^9^ FOXP3+Tregs are an immunosuppressive subset of CD4+T cells, with FOXP3 representing a master transcription factor that modulates the development and functions of these cells.^36^ High Treg infiltration suggests that OTSCC are poised to respond to immunotherapeutic modalities that relieve inhibitory pathways, and of clinical relevance CTLA4 is a target of the FOXP3 transcription factor.^37^, ^38^ Furthermore, although there were generally little differences in IHC immune profiles between less and more advanced disease we noted that Programmed cell death 1 ligand‐1 (PD‐L1) TPS assessed by two clinically relevant antibodies was shown to be significantly elevated in OTSCC cases (*P* < .001), and recurrent OTSCC cases (*P* = .004 by SP263, Ventana and *P* = .019 by 22C3, Dako). This confirms firstly that therapeutic targeting of the PD‐1/PD‐L1 axis in HNSCC is a potential treatment option which has been confirmed in multiple landmark studies using PD‐1/PD‐L1 inhibitors in biomarker selected and unselected patients.^1^, ^2^ However, the modest overall response rates with monotherapy and the controversial usefulness of PD‐L1 expression as a predictive biomarker, emphasizes the importance of further research into combination therapy approaches and more reliable biomarkers for patient selection.^1^, ^2^, ^11^ Indeed, our data provide a molecular rationale for the limited success of immunotherapy used as monotherapy in OTSCC given presence of likely primary immune failure, and the lack of dynamic changes observed in tumors with more advanced disease. Numerous trials are exploring immunotherapy combination approaches in advanced cancers with a backbone of PD‐1/PD‐L1/CTLA4 inhibition in conjunction with Treg cell focused agents including those targeting the Inducible T cell Co‐Stimulator (ICOS) receptor (NCT02723955), and GITR (NCT03126110). However, it may be that these immunotherapy approaches or combined chemo‐immunotherapy approaches need to be introduced much earlier in treatment algorithms before further major breakthroughs are achieved in OTSCC.

Our study was limited by the relatively small number of 110 FFPE samples from 67 OTSCC cases analyzed. However, given our comprehensive analyzes within a well annotated, homogeneous OTSCC cohort we have demonstrated the usefulness of uniform pilot cohorts enriched for mechanisms not apparent in heterogeneous cohorts. Another important consideration of this study was the utilization of FFPE tumor samples for analyzes that contained mixtures of tumoral, epithelial cells, stromal cells, and immune cells. This was a deliberate aspect of the study design in order to appreciate the impact of the tumor microenvironment as a whole. In addition, our cohort of OTSCC patients were not treated with immunotherapy and further usefulness of the findings requires prospective validation in larger, immunotherapy treated, homogeneous cohorts of HNSCC patients.

## 5 CONCLUSION

We identified a 24‐immune gene signature in OTSCC that had features of a TH‐2‐type inflammatory response that was not associated with survival for HNSCC but was prognostic in other cancer types, highlighting the presence of common pathways impacting patient outcome with histotype‐dependent effects. We identified primary immune failure to prevent carcinogenesis in OTSCC being dominated by immunosuppressive signaling, with the absence of immune response evolution to the development of more advanced disease, emphasizing the need for early combination therapeutic approaches.

## CONFLICT OF INTEREST

AM Lim ‐ uncompensated advisory board from Merck Sharp & Dohme and Bristol‐Myers Squibb with travel and accommodation expenses. The other authors declare that there is no potential conflict of interest.

## AUTHOR CONTRIBUTIONS

KM and AML were involved in study concept, study design, study funding, data analyses, and manuscript drafting; KM, BM, YK, CS, PF, and AML were involved in data collection; KM, CL, BM, JL, NCW, GMA, TS, and AML were involved in laboratory analyses; KM, CL, ML, AJ, MB, NCW, BS, and AML were involved in data and statistical analyses. All authors were involved in manuscript review and approval.

## Supporting information

Figure S1‐S5

Table S1‐S6

File S1

File S2

File S3

File S4

## Data Availability

Expression data generated for this manuscript are available in the Gene Expression Omnibus data repository (GSE148944).
