# Supplementary material for: Characterization of the immune profile of oral tongue squamous cell carcinomas with advancing disease
Source: Cancer Med. 2020 May 8;9(13):4791–807. doi: 10.1002/cam4.3106 (PMC7333861; doi:10.1002/cam4.3106)

**SUPPLEMENTARY FIGURES**

**Supplementary Figure 1.** Representative image of intra- and peri-tumoral annotations. The red outlines the intra-tumoral area and the green outlines the peri-tumoral region.


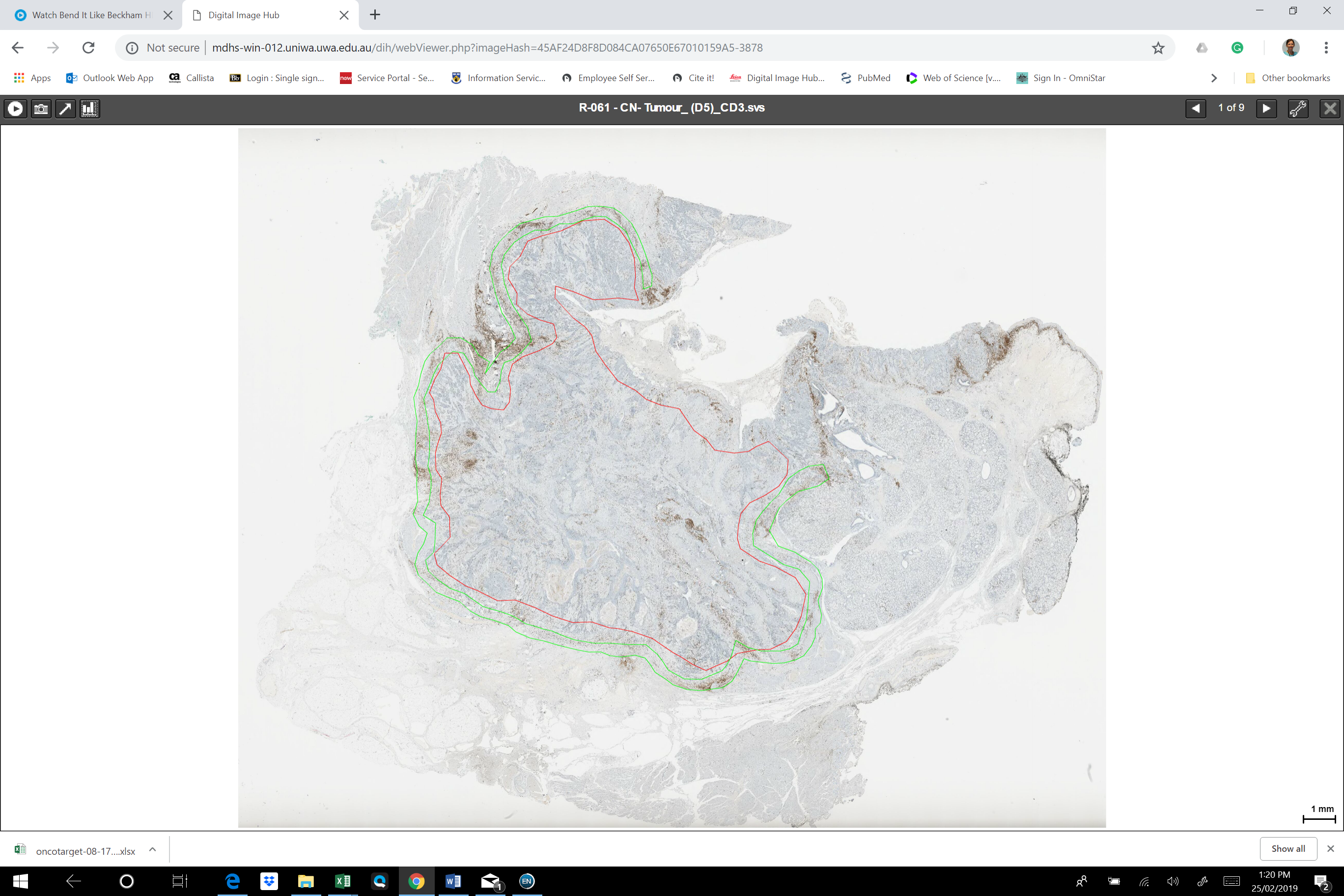


**Supplementary Figure 2.** KM plotter Kaplan Meier curves in other cancer types with FDR< 5% for specific genes of interest identified in our OTSCC cohort (kmplot.com) (22).


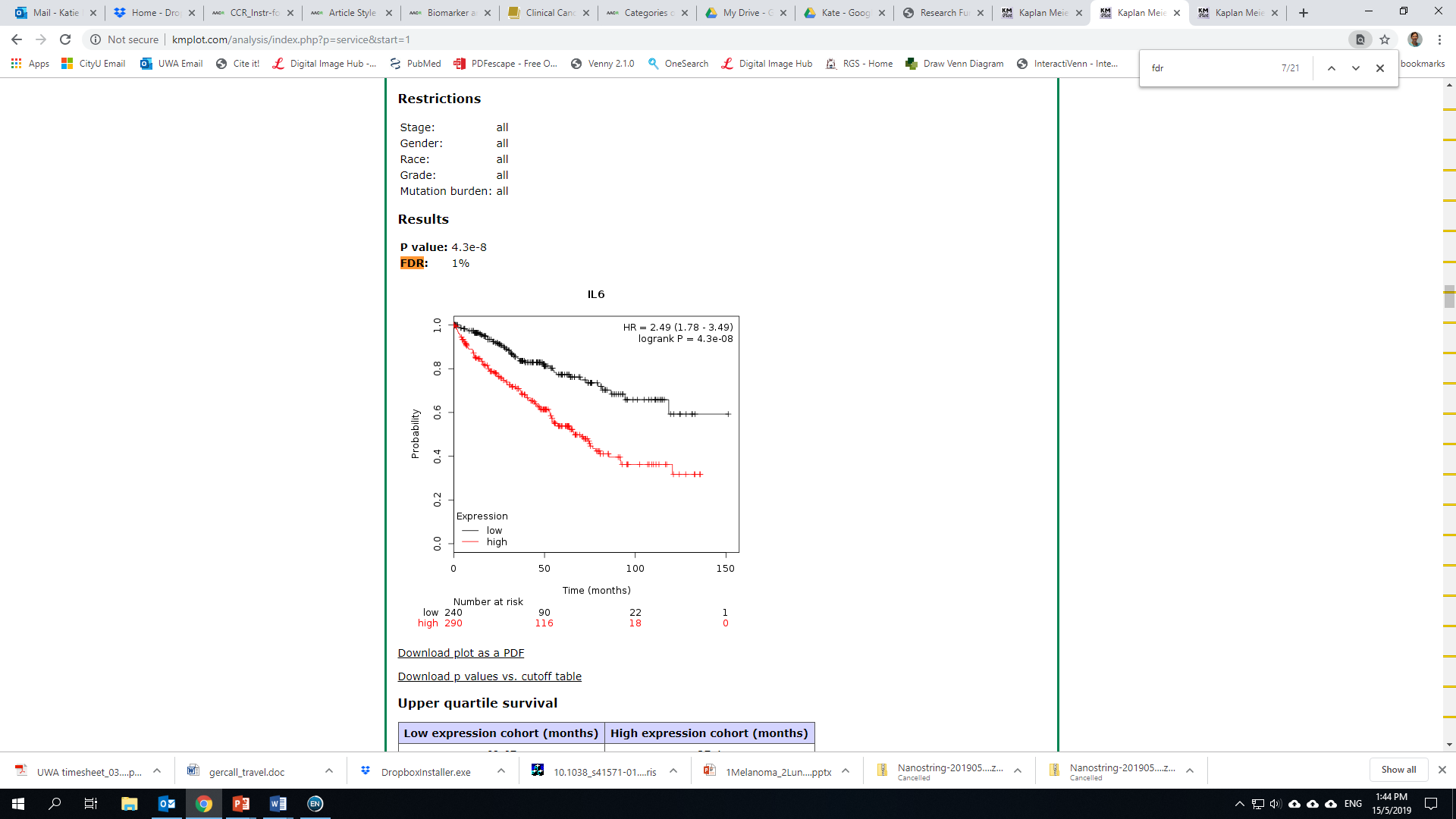


Renal clear cell carcinoma (FDR 1%, median survival 70 vs 27 months for those with low and high IL6 expression respectively).


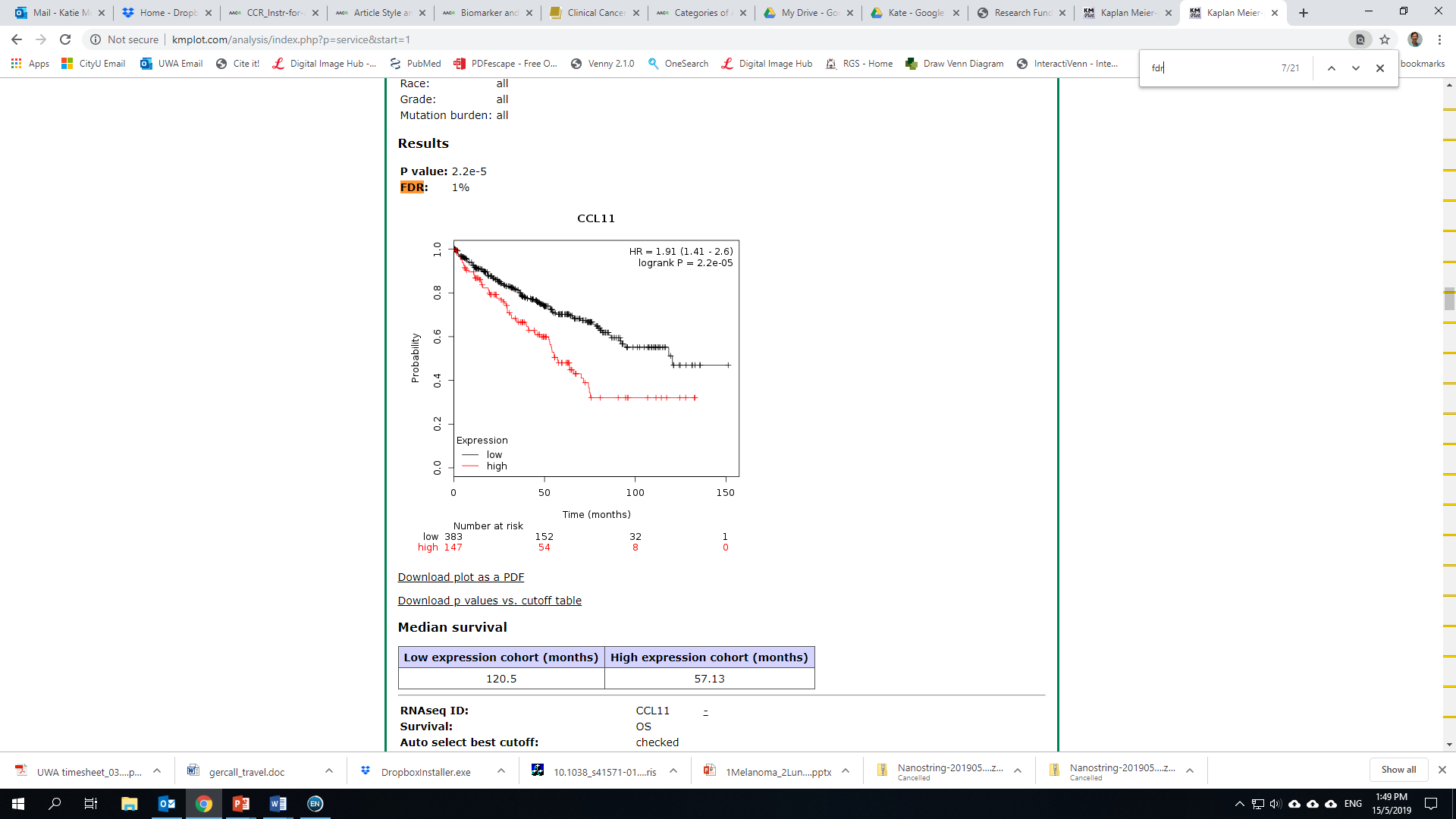


Renal clear cell carcinoma (FDR 1%, median survival 120 vs 57 months for those with low and high CCL11 expression respectively).


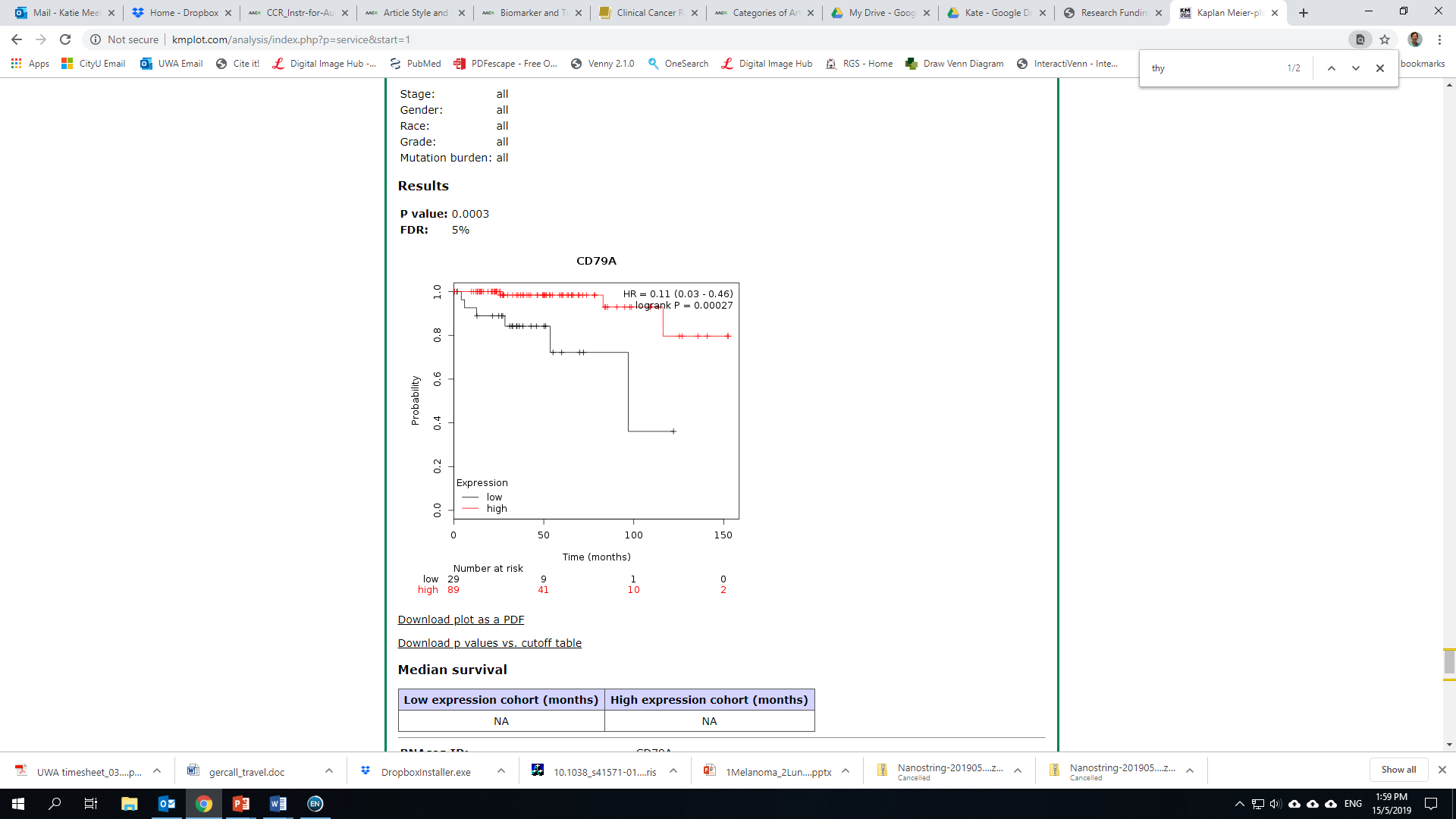


Thymoma (FDR 1%, median survival for CD79A not available).

Renal clear cell carcinoma (FDR 1%, median survival 70 vs 28 months for those with low and high combined IL6/CCL11 expression respectively).


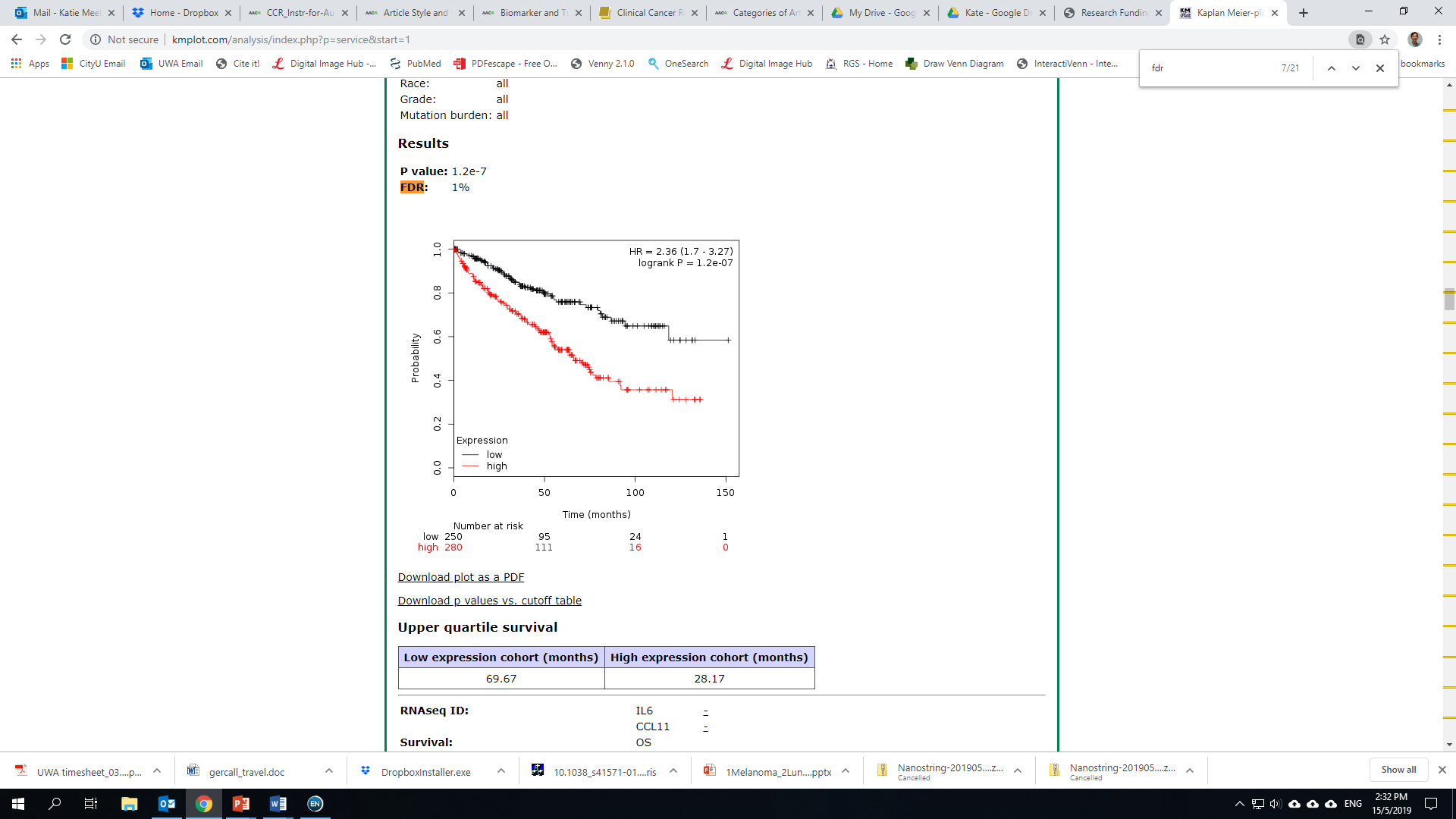


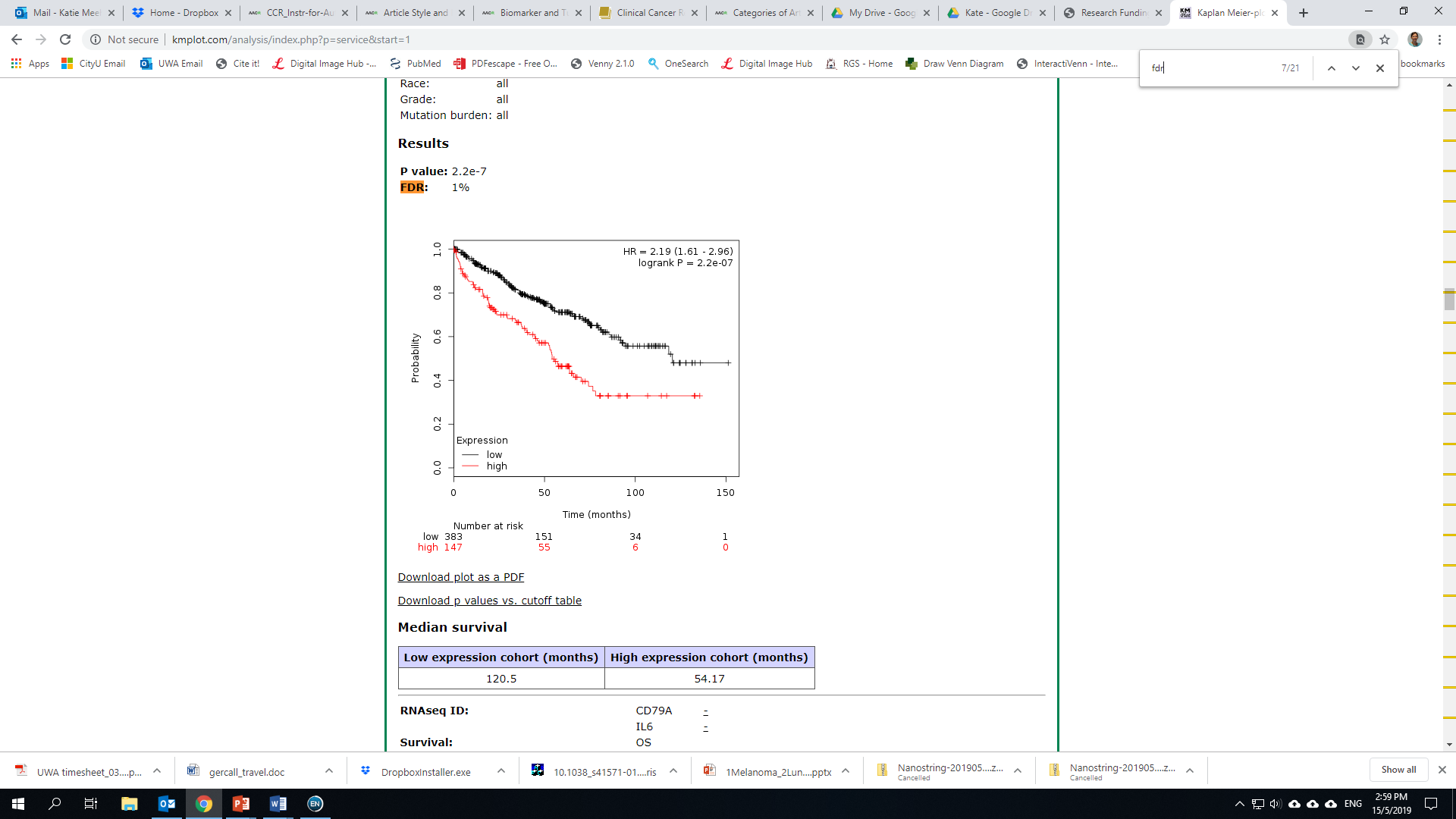
**Supplementary Figure 3.** Representative images of PD-L1 expression in recurrent OTSCC and non-recurrent disease. Histology shown by H&E (images labelled A) and higher expression of PD-L1 assessed by the SP263 clone in recurrent tumors (image B, panel B) compared with non-recurrent tumors (image B, panel A).


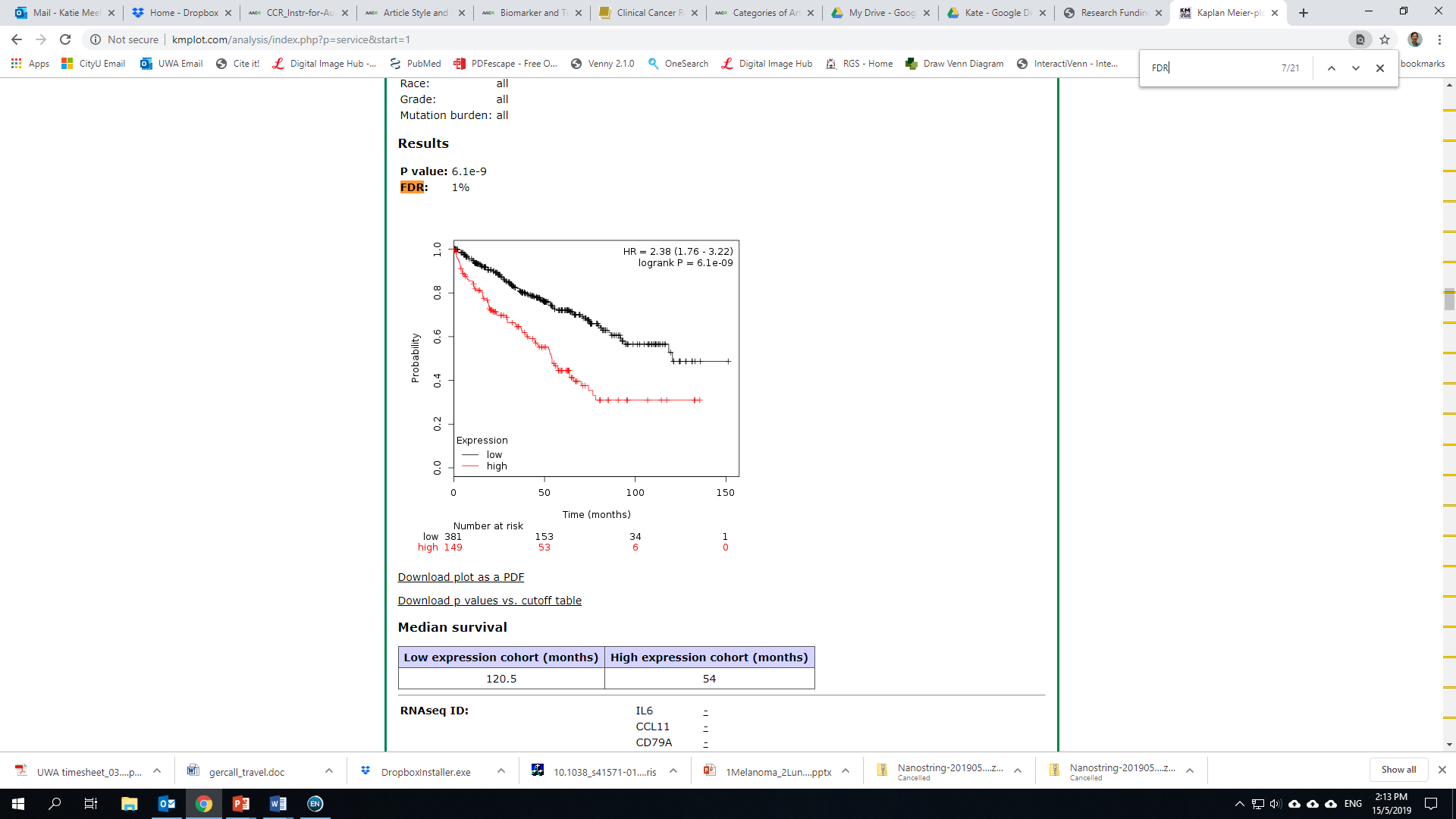


Renal clear cell carcinoma (FDR 1%, median survival 120 vs 54 months for those with low and high combined CD79A/CCL11/IL6 expression respectively).

Renal clear cell carcinoma (FDR 1%, median survival 120 vs 54 months for those with low and high combined CD79A/IL6 expression respectively).


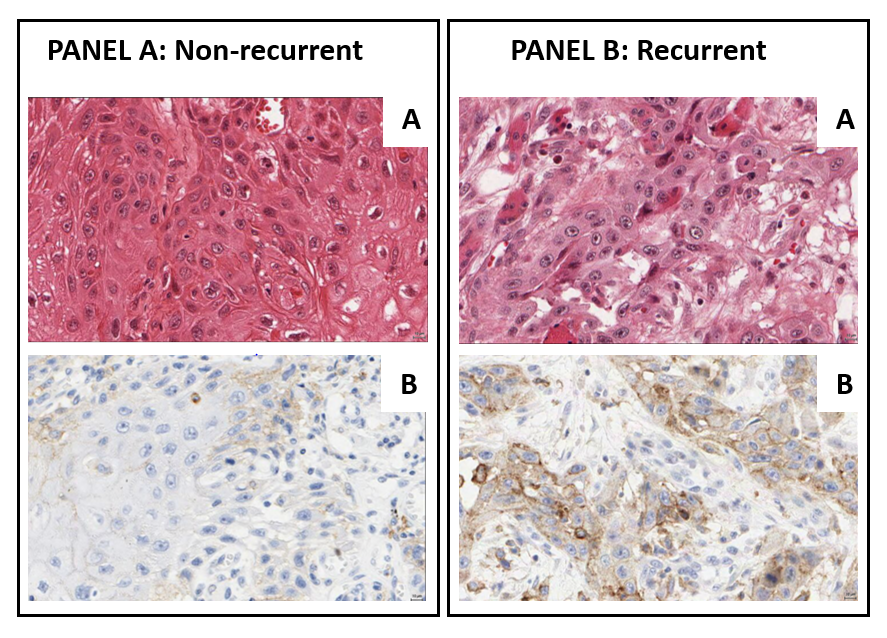


**Supplementary Figure 4.** Representative images of overexpressed immune markers in OTSCC tumor specimens compared with matched, involved lymph nodes. Histology shown by H&E (images labelled A) and similar expression of CD3 (B), CD4 (C) and higher expression of CD56 (D) in tumor (Panel A) compared with matched lymph node (Panel B).

*
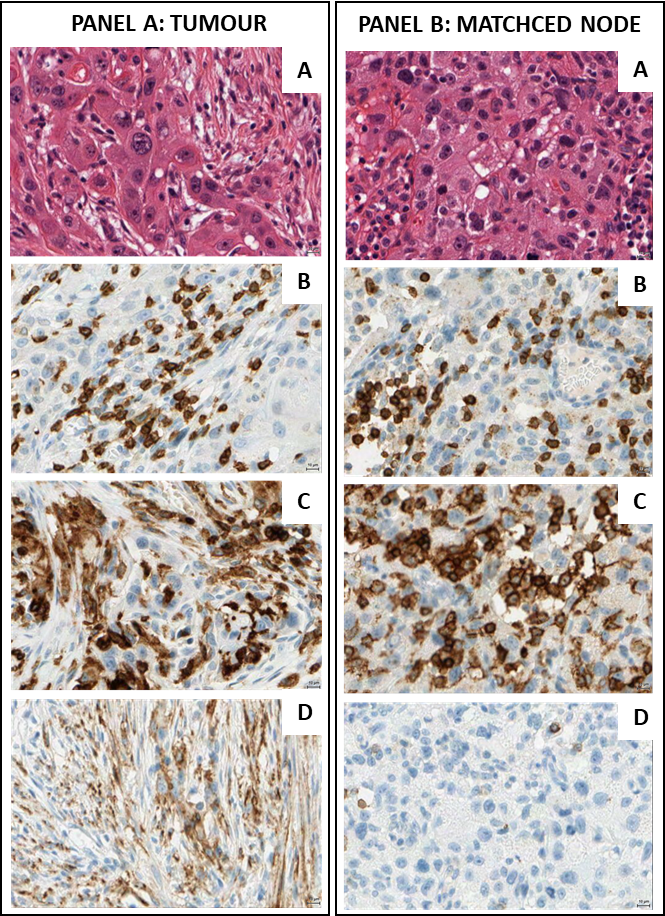
*

**Supplementary Figure 5.** Schematic showing genes involved in the cytokine-cytokine receptor interaction pathway (red nodes) (20,21). The lines joining each gene represent the level of evidence supporting the interaction. Light blue and purple lines demonstrate known interactions based on experimental evidence or curated database information. Green lines represent an interaction based on text mining and black represents co-expression.


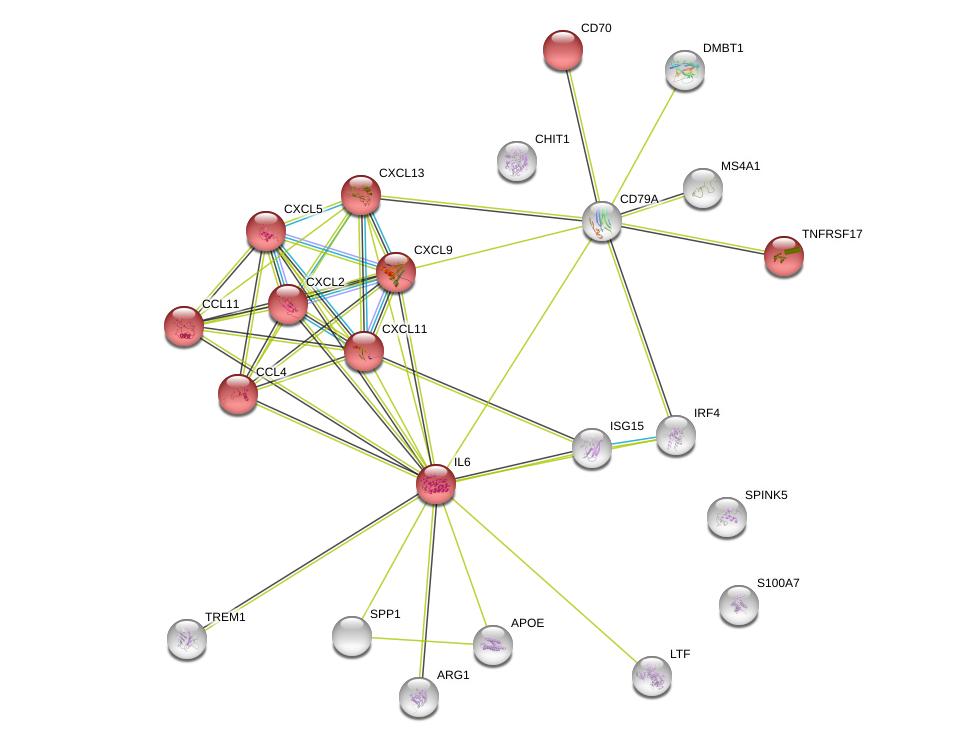

Supplement: Supplementary file 1 — Figure S1‐S5 [file CAM4-9-4791-s001.docx]
