# Supplementary material for: Characterization of the immune profile of oral tongue squamous cell carcinomas with advancing disease
Source: Cancer Med. 2020 May 8;9(13):4791–807. doi: 10.1002/cam4.3106 (PMC7333861; doi:10.1002/cam4.3106)
Supplement: Supplementary file 2 — Table S1‐S6 [file CAM4-9-4791-s002.docx]

**SUPPLEMENTARY TABLES**

**Supplementary Table 1.** Clinicopathological features of the OTSCC cohort.

|  |  | N=67 (%) |
| --- | --- | --- |
| **Follow up time** *Med [Min-max]* |  | 31 [1-259] |
| **Age** *Med [Min-max]* |  | 59.4 [18.8-89.5] |
| **Gender** | F | 35 (52.2) |
|  | M | 32 (47.8) |
| **Stage** | I | 25 (37.3) |
| (AJCC 7^th^) | II | 11 (16.4) |
|  | III | 10 (14.9) |
|  | IV | 21 (31.3) |
| **T-category** | 1 | 27 (40.3) |
|  | 2 | 20 (29.9) |
|  | 3 | 13 (19.4) |
|  | 4 | 7 (10.4) |
| **N-category** | 0 | 41 (61.2) |
|  | 1 | 8 (11.9) |
|  | 2 | 13 (19.4) |
|  | 3 | 5 (7.5) |
| **M-category** | 0 | 67 (100.0) |
| **Histopathological Grade** | Low /Well differentiated | 16 (23.9) |
|  | Intermediate/Moderately differentiated | 39 (58.2) |
|  | High/Poorly differentiated | 12 (17.9) |
| **Depth of invasion** | <5mm | 14 (20.9) |
|  | 5-10mm | 19 (28.4) |
|  | >10mm | 17 (25.4) |
|  | Unknown/missing | 17 (25.4) |
| **Smoking history** | No | 18 (26.9) |
|  | Yes | 39 (58.2) |
|  | Unknown | 10 (14.9) |
| **Alcohol intake** | Non-drinker | 13 (19.4) |
|  | Light | 19 (28.4) |
|  | Moderate | 8 (11.9) |
|  | Heavy | 11 (16.4) |
|  | Unknown/missing | 17 (25.4) |

**Supplementary Table 2.** PD-L1 staining comparison of SP263 Ventana antibody versus the 22C3 Dako antibody. Among the 80 cases examined, 72% (58/80) and 57% (46/80) were found to express PD-L1 using the Ventana and Dako antibodies, respectively. T-tests demonstrated that both antibodies gave similar results for all comparison groups (p>0.05) despite fewer cases being stained using the Dako antibody.

| **Group** | **Antibody** | **N** | **Mean PD-L1 TPS (%)** | **Std. Dev** | **TPS Range (%)** | **t-test** | **P-value**  **(2-tailed)** |
| --- | --- | --- | --- | --- | --- | --- | --- |
| Non-Cancer cases | Ventana | 10 | 0.9 | 1.6 | 0-5 | 0.943 | 0.358 |
|  | Dako | 10 | 0.4 | 0.5 | 0-1 |  |  |
| OTSCC | Ventana | 78 | 19.3 | 27.3 | 0-95 | 0.798 | 0.426 |
|  | Dako | 78 | 15.9 | 25.9 | 0-90 |  |  |
| OTSCC without Nodes | Ventana | 32 | 6.7 | 8.6 | 0-30 | 1.136 | 0.260 |
|  | Dako | 32 | 4.3 | 7.8 | 0-30 |  |  |
| OTSCC with nodal involvement | Ventana | 18 | 20.3 | 29.6 | 0-80 | -0.011 | 0.991 |
|  | Dako | 18 | 20.4 | 30.6 | 0-90 |  |  |
| Matched, involved nodes | Ventana | 18 | 7.5 | 11.4 | 0-40 | -0.048 | 0.962 |
|  | Dako | 18 | 7.7 | 16.1 | 0-50 |  |  |
| Recurrent OTSCC | Ventana | 29 | 33.9 | 33.1 | 0-95 | -1.055 | 0.296 |
|  | Dako | 27 | 24.9 | 31.0 | 0-95 |  |  |
| OTSCC without recurrence | Ventana | 50 | 11.6 | 19.8 | 0-80 | 0.790 | 0.433 |
|  | Dako | 50 | 10.1 | 20.6 | 0-90 | 0.366 | 0.715 |

TPS – tumor positive score

**Supplementary Table 3.** NanoStringDiff R code. Transcript counts were normalized using the NanoStringDiff package within Bioconductor, which involved estimating parameters from positive controls, negative controls, and housekeeping genes embedded in the nCounter system. Differential expressions of genes were assessed using log2-normalised data with a generalized linear model likelihood ratio test, using the glm.LRT function within the NanoStringDiff package.

| NanoStringDiff R code |
| --- |
| library("Biobase")  library("NanoStringDiff") |
| **Comparison 1 = Cancer vs Non-Cancer**  directory <- system.file("extdata", package="NanoStringDiff", mustWork=TRUE)  C1<-paste(directory,"c1.csv",sep="/")  designsc1=data.frame(group=c("C", "C","C", "C", "C", "C", "C", "C", "C", "C", "C","C", "C", "C","C", "C","C", "C", "C", "C", "C", "C", "C", "C", "C","C", "C", "C","C", "C","C", "C", "C", "C", "C", "C", "C", "C", "C","C", "NC","NC","NC","NC","NC","NC"))  designsc1  library("NanoStringDiff")  C1data=createNanoStringSetFromCsv(C1,header=TRUE,designsc1)  C1data  pData(C1data)  head(exprs(C1data))  pheno=pData(C1data)  group=pheno$group  design.full=model.matrix(~0+group)  design.fullC1  contrastC1=c(-1,1)  C1data1=estNormalizationFactors(C1data)  positiveFactor(C1data)  negativeFactor(C1data)  housekeepingFactor(C1data)  COMP1result=glm.LRT(C1data1,design.fullC1,contrast=contrastC1)  head(COMP1result$table)  str(COMP1result) |
| **Comparison 2 = OTSCC with nodal involvement vs OTSCC without nodal involvement**  directory <- system.file("extdata", package="NanoStringDiff", mustWork=TRUE)  Comparison2<-paste(directory,"c2.csv",sep="/")  designsCOMP2=data.frame(group=c("WN", "WN","WN", "WN", "WN", "WN", "WN", "WN", "WN", "WN", "WN", "WN", "WN", "WN", "NN", "NN", "NN", "NN", "NN", "NN", "NN", "NN", "NN", "NN", "NN", "NN", "NN","NN"))  designsCOMP2  library("NanoStringDiff")  COMP2data=createNanoStringSetFromCsv(Comparison2,header=TRUE,designsCOMP2)  COMP2data  pData(COMP2data)  head(exprs(COMP2data))  pheno=pData(COMP2data)  group=pheno$group  design.full=model.matrix(~0+group)  design.fullC2  contrastCOMP2=c(-1,1)  COMP2data1=estNormalizationFactors(COMP2data)  positiveFactor(COMP2data)  negativeFactor(COMP2data)  housekeepingFactor(COMP2data)  COMP2result=glm.LRT(COMP2data1,design.fullC2,contrast=contrastCOMP2)  head(COMP2result$table)  str(COMP2result) |
| **Comparison 3 = Recurrent OTSCC vs non-recurrent OTSCC**  directory <- system.file("extdata", package="NanoStringDiff", mustWork=TRUE)  C3<-paste(directory,"c3.csv",sep="/")  DesC3=data.frame(group=c("NR","NR","NR","NR","NR","NR","NR","NR","NR","NR","NR","NR","NR","NR","NR","NR","NR","NR","NR","NR","NR","NR","NR","NR","NR","NR","NR","NR","RC","RC","RC","RC","RC","RC","RC","RC","RC","RC","RC","RC"))  DesC3  library("NanoStringDiff")  C3data=createNanoStringSetFromCsv(C3,header=TRUE,DesC3)  C3data  pData(C3data)  head(exprs(C3data))  phenoC3=pData(C3data)  group=phenoC3$group  design.fullC3=model.matrix(~0+group)  design.fullC3  contrastC3=c(-1,1)  C3data1=estNormalizationFactors(C3data)  positiveFactor(C3data)  negativeFactor(C3data)  housekeepingFactor(C3data)  COMP3result=glm.LRT(C3data1,design.fullC3,contrast=contrastC3)  head(COMP3result$table)  str(COMP3result) |
| **Comparison 4 = Primary Tumour vs Tumour in involved, matched Lymph Node**  directory <- system.file("extdata", package="NanoStringDiff", mustWork=TRUE)  C4<-paste(directory,"c4.csv",sep="/")  DesC4=data.frame(group=c("T","T","T","T","T","T","T","T","T","T","T","T","T","T","T","T","T","T","N","N","N","N","N","N","N","N","N","N","N","N","N","N","N","N","N"))  DesC4  library("NanoStringDiff")  C4data=createNanoStringSetFromCsv(C4,header=TRUE,DesC3)  C4data  pData(C4data)  head(exprs(C4data))  phenoC4=pData(C4data)  group=phenoC4$group  design.fullC4=model.matrix(~0+group)  design.fullC4  contrastC4=c(-1,1)  C4data1=estNormalizationFactors(C4data)  positiveFactor(C4data)  negativeFactor(C4data)  housekeepingFactor(C4data)  COMP4result=glm.LRT(C4data1,design.fullC4,contrast=contrastC4)  head(COMP4result$table)  str(COMP4result) |

**Supplementary Table 4**. Additional statistical summary for *in silico* validation.

| *In silico* validation using the TCGA cohorts was performed in three stages. Firstly, the sum of the four down regulated genes for each patient (patient mean) was calculated and compared to the cohort mean for these genes. If patient sum was below the cohort mean, a binary value of ‘1’ was assigned to designate this case as having the cancer signature. If the patient sum was greater than the cohort mean, a binary value of ‘0’ was assigned to designate this case as being negative for the cancer signature. Secondly, the sum of 19 upregulated genes for each patient was calculated and compared to the cohort mean for these genes. If the patient sum was greater than the cohort mean, a binary value of ‘1’ was assigned to designate this case as having the cancer signature. If the patient sum was below the cohort mean, a binary value of ‘0’ was assigned to designate this case as being negative for the cancer signature. Finally, cases were scored as having the ‘full cancer signature’ if they were assigned 1/1 values from stages 1 and 2 described above. Cases with 0/1 or 1/0 were described as having “part of the cancer signature” and 0/0 values were scored as not having the cancer signature. |
| --- |

**Supplementary Table 5**. Statistical explanation for logistic regression models.

| ***Full HNSCC TCGA cohort:*** *Linearity of the genes in the MV model with respect to the logit of the outcome variable (death) was assessed via the Box-Tidwell procedure (none were significant at p<0,05 so linearity of IVs was assumed). MV model#1 was built taking into account both the univariate* significant genes (at p<0.015) as candidate predictors (as per standard model building techniques) and genes significant in an initial MV model** (using all genes as candidate predictors in order to take into account any synergistic gene effects). A Bonferroni correction was applied to the alpha level in the MV models resulting in an adjusted alpha (p<0.05/4=0.0125) for a 4-covariate model. APOE was not significant in MV model#1 at p<0.0125, after the Bonferroni correction was applied. Diagnostics for collinearity in the final model were based on calculation of variance inflation factors (VIF), derived from the coefficient of determination (R^2^), for all predictors regressed against each other, where VIF=1/(1-R^2^). VIF>2.5 was considered high (all covariate VIFs were <1.2). Final MV model was based on genes that were collectively significant and met final assumptions for collinearity and alpha<0.05/3=0.0167 (for a 3-covariate model), after a Bonferroni adjustment. ROC AUC estimates were computed for all genes in the final logistic model in order to assess discrimination for the model. ROC curve analysis (based on logistic models) for all 3 genes combined (CD79A, IL6, CCL11) AUC=0.632 and individual gene model AUC’s were CD79A=0.601, IL6 =0.537 and CCL11=0.563.* |
| --- |
| ***OTSCC TCGA subgroup:*** *Linearity of the 5 genes in the MV model #1 with respect to the logit of the outcome variable (death) was assessed via the Box-Tidwell procedure (none were significant at p<0,05 so linearity of IVs was assumed). MV model#1 was built taking into account both the univariately significant genes (at p<0.015) as candidate predictors (as per standard model building techniques) and genes significant in an initial MV model (using all genes as candidate predictors in order to take into account any synergistic gene effects). A Bonferroni correction was applied to the alpha level in the MV models resulting in an adjusted alpha (p<0.05/2=0.025) for a 2-covariate model. Diagnostics for collinearity in the final model were based on calculation of variance inflation factors (VIF), derived from the coefficient of determination (R2), for all predictors regressed against each other, where VIF=1/(1-R2). VIF>2.5 was considered high (VIF was<1.31). Final MV model was based on genes that were collectively significant and met final assumptions for collinearity and after Bonferroni adjustment.* |

| **Gene** |  |  | **Degust** | | **NSolver** | **NanoDiff** | |
| --- | --- | --- | --- | --- | --- | --- | --- |
|  | Average Log FC | StDev | P- value | FDR | P-value | P-value | Q-value |
| *CXCL9* | 1.59 | 0.04 | <0.001 | 0.024 | 0.008 | <0.001 | 0.025 |
| *DMBT1* | -2.88 | 0.31 | 0.024 | 0.042 | 0.019 | ND | ND |

**Supplementary Table 6**. Differentially expressed genes in recurrent OTSCC cancer versus non-recurrent cases.

FDR-false discovery rate; ND-not detected; StDev-standard deviation
